# Day case hip and knee replacement in England: a population-based cohort study using linked National Joint Registry and Hospital Episode Statistics data

**DOI:** 10.1186/s12916-025-04280-y

**Published:** 2025-10-14

**Authors:** Jonathan M. R. French, Kevin Deere, Adrian Sayers, Michael R. Whitehouse

**Affiliations:** 1 Musculoskeletal Research Unit, Learning and Research Building, Southmead Hospital, University of Bristol Medical School, Bristol, BS7 0UL UK; 2 National Institute for Health Research Bristol Biomedical Research Centre, University Hospitals Bristol and Weston NHS Foundation Trust and University of Bristol, Bristol, UK

**Keywords:** Day case, Same day, Outpatient, Ambulatory, Joint replacement, Hip replacement, Knee replacement, Arthroplasty

## Abstract

**Background:**

In response to rising demand, disruptions from the COVID-19 pandemic, and the need for improved cost-effectiveness, the way hip and knee replacements are being delivered is rapidly changing. Increasingly, they are being performed as day case procedures without an overnight stay in hospital. This study assessed the safety of this for a national cohort of NHS-funded procedures in England.

**Methods:**

Data from the National Joint Registry, Hospital Episode Statistics, and Civil Registration of Deaths databases were linked to identify patients who underwent total hip replacement (THR) and total or unicompartmental knee replacement (TKR/UKR) in England between 2010 and 2022. Outcomes including 30-day readmissions, 90-day serious adverse events, and 1-year reoperations were compared between day case and one-day stay inpatients using adjusted flexible parametric survival models.

**Results:**

The study included 7485 day case and 60,747 one-day stay inpatient procedures. Day case surgery was associated with a higher risk of 30-day readmission for THR (adjusted relative risk (RR) 1.28, 95% CI 1.07 to 1.53) and TKR (RR 1.28, 95% CI 1.10 to 1.48). A learning curve was observed where the first 6-day case THRs and the first 4-day case TKRs per unit carried significantly higher readmission risk. There were no differences in 90-day serious adverse events. However, day case TKR was associated with an increased risk of reoperation within 1 year (RR 1.50, 95% CI 1.15 to 1.96; NNTH 84), most commonly manipulation under anaesthesia (MUA). No significant differences were found for UKR.

**Conclusions:**

Day case UKR appears safe. Day case THR is generally safe, and although there is a higher risk of readmission in the first six procedures at each unit, other safety outcomes are not different. However, day case TKR carries an increased risk of reoperation, mainly for MUA which is typically performed for postoperative stiffness.

**Supplementary Information:**

The online version contains supplementary material available at 10.1186/s12916-025-04280-y.

## Background

Hip and knee replacements are common and highly effective operations to treat end-stage arthritis [1, 2]. Globally, over two million are performed per year [3, 4]. The incidence is projected to rise significantly as populations age [5], a burden further compounded by the effects of the COVID-19 pandemic, where disruption to routine hospital services incurred unprecedented losses of joint replacement activity across many countries [6, 7]. 

In response to this, the way hip and knee replacements are delivered has been fundamentally changing. Increasingly, they are being performed as day cases, where patients do not have an overnight stay in hospital [8, 9]. The practice was increasing in popularity in the US prior to the pandemic due to changes in financial reimbursement, resulting in more than half of hip and knee replacements now being performed as day cases [10]. In Canada, the circumstances surrounding the pandemic and its aftermath have culminated in an approximately 100-fold relative increase in the practice [7]. In England, a 2023 Getting It Right First Time (GIRFT) initiative issued guidance recommending a default length of stay for hip and knee replacement of 0 to 1 day [11].

Performing major procedures as day case to optimise resource utilisation may carry the risk of unintended consequences such as change in risk of unplanned admissions or adverse events. Whilst existing evidence appears reassuring, it predominantly comprises small, low-quality studies with significant risks of bias, limited follow-up, and uncertain generalisability beyond the US [12]. UK data on the practice remains sparse [12, 13]. In light of the planned substantial shift in practice, robust evidence is urgently needed in a previously identified James Lind Alliance top 10 research priority area [14].

We aimed to apply modern epidemiological methods to routinely collected, linked national datasets, which have mandatory data submission, to compare outcomes following NHS-funded day case total hip replacement (THR), total knee replacement (TKR), and unicompartmental (partial) knee replacement (UKR) in England, to those following a 1-day inpatient stay.

## Methods

### Study design and data sources

**Fig. 1:**
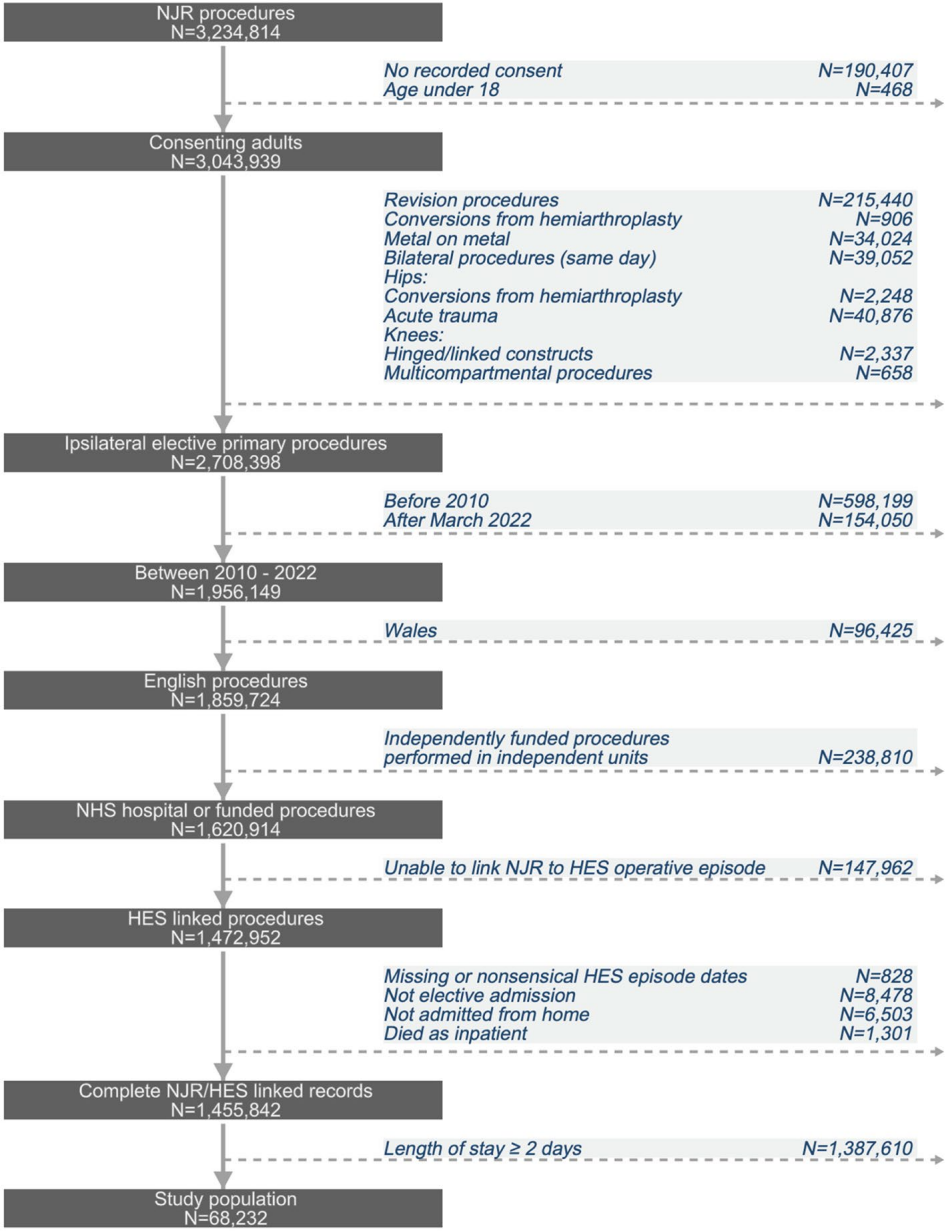
Data flowchart for linked National Joint Registry (NJR) and Hospital Episode Statistics (HES) hip and knee replacements

This was a population-based prospective cohort study using linked, routinely collected data for hip and knee replacements undertaken at public hospitals, and publicly funded procedures in independent hospitals in England from 1 st January 2010 to 31 st March 2022. Data from the National Joint Registry (NJR) of England were linked to the NHS Hospital Episode Statistics (HES) database and to civil registration mortality data. Data access was granted after application through the NJR Research Committee. Data submission to the NJR is mandatory for all hip and knee replacements and includes patient, surgeon, and operation details. Annual data quality audits consistently show > 96% completeness of all primary hip and knee data [15]. HES records all NHS-funded activity in both NHS and independent units in England, and includes length of stay, demographic, diagnosis, procedural, and administrative data. HES data are used for the accurate reimbursement of NHS providers for their activities and for the purposes of this study required cleaning into ‘superspells’ [16] using the process outlined in Additional file 1: Figure S1. HES linkage was successful in 90.9% of procedures (Fig. 1).

### Selection criteria

All patients aged 18 years and older having an elective primary hip or knee replacement were eligible for inclusion. Three groups of operations were considered separately: THR, TKR, and UKR. Exclusion criteria were lack of NJR patient consent, reason for surgery recorded as acute trauma or cancer, same-day bilateral procedures, missing or non-sensical HES episode data, non-elective admissions, patients not admitted from home, patients who died in hospital, and patients who stayed more than one night in hospital (Fig. 1).

### Exposure

The exposure was day case surgery, i.e. the patient was admitted, operated on, and discharged on the same calendar date. The comparison group was 1-day stay inpatient procedures, i.e. the patient was operated on then discharged home the next calendar day. One-day stay inpatients are a more valid comparison group than the broader inpatient population for assessing day case surgery [17], and have greater similarity in their demographic characteristics (Additional file 1: Figures S2 to S4).

### Outcomes

The primary outcome was 30-day readmissions, defined as unplanned admission to any hospital from the patient’s usual residence. Admissions with an ‘emergency’ HES admission method code were considered unplanned (Additional file 1: Figure S1).

The secondary outcomes were 90-day serious adverse events, 1-year reoperations, and risk factors for each outcome. Serious adverse events were defined as admissions to hospital due to the following medical complications, identified using ICD-10 (International Classification of Diseases, 10th revision) codes: venous thromboembolism (VTE; pulmonary embolism [PE] or deep vein thrombosis [DVT]), myocardial infarction (MI), pneumonia, urinary tract infection (UTI), stroke (Additional file 1: Figure S5), or all-cause death. Reoperation included both revision (where any implant that is part of the joint replacement construct is added, removed or modified, recorded in the NJR) and non-revision procedures, identified using OPCS-4 codes from HES (Additional file 1: Figure S6).

### Statistical analysis

Statistical analysis used flexible parametric survival models including day case surgery as a time-varying covariate. Restricted cubic splines were used to allow for modelling nonlinearity in the baseline hazard function. This allowed evaluation of both relative and absolute risks for adverse outcomes and their timings. The best fitting unadjusted model was selected with the most parsimonious number of knots using the Akaike Information Criterion (AIC), Bayesian Information Criterion (BIC), and visual inspection of plots. Models were then adjusted for patient, socioeconomic, surgical, and unit characteristics decided a priori to be of potential clinical importance. Patient characteristics were age, sex, American Society of Anaesthesiologists (ASA) grade, Charlson Comorbidity Index, obesity, mental health diagnosis, smoking, number of hospital admissions in the year prior to surgery, and previous contralateral joint replacement. Socioeconomic characteristics were ethnicity, Index of Multiple Deprivation (IMD) quintile, and rural or urban residence. Surgical characteristics were indication, approach, component fixation, bearing materials (THR), bearing design (TKR, UKR), femoral head size (THR only), compartment (UKR only), anaesthetic, and VTE prophylaxis. Unit characteristics were sector (NHS or independent), cumulative day case procedure volume, and date of surgery. Further details of each covariate can be found in Additional file 1: Figure S7. Multicollinearity was tested for using variance inflation factors with a cutoff of 5.

Continuous variables were modelled using restricted cubic splines to account for non-linear associations. Risk factors for adverse outcomes with day case surgery specifically were identified by including each variable as an interaction term. Model fit was tested using likelihood ratios, and significant interactions were incorporated into the final model. Adjusted relative and absolute risks between groups were generated using standardised survival probabilities [18], with covariates standardised to.

those observed in the day case population, giving the average treatment effect of day case surgery for patients as they were empirically selected.

### Missing data and sensitivity analyses

The NJR data had been prepared for this analysis as described in the NJR 2023 20th Annual Report, which included prior removal of records with missing information, duplicate procedures, and records where a logical sequence of primary and revision procedures was unable to be ascertained [19] (Additional file 1: Figures S8, S9). Body mass index (BMI) was missing in 18% of NJR cases and was therefore converted to binary obesity with a cutoff of ≥ 30 kg/m^2^, with missing values imputed using presence or absence of obesity ICD-10 codes from HES. IMD and type of residence were missing in 0.6% of cases. These and cases with an ASA grade of IV–V, due to their rarity (*n* = 3 day cases), were excluded from adjusted analyses. A sensitivity analysis was performed to test for differences in univariable model output with and without these cases. Further sensitivity analyses involved testing for clustering by unit and surgeon using mixed-effect multilevel logistic regression modelling. The validity of using 1-day stay inpatients as the comparison group, rather than all inpatients, was assessed by inspecting long-term crude readmission and mortality rates. All analyses were performed using Stata (version 18, StataCorp, USA).

### Patient and public involvement

Optimising peri-operative protocols was specified as a priority in two of the top 10 research priorities identified by patients and clinicians from the 2014 James Lind Alliance Priority Setting Partnership on hip and knee replacement for osteoarthritis [14]. Patient representatives sit on the NJR research committee and provided feedback during the data access approval process. Advocacy efforts in the UK such as Versus Arthritis’ ‘Impossible to Ignore’ petition have been signed by tens of thousands of patients asking for government action to tackle the post-pandemic joint replacement backlog; increasing day case surgery is part of the NHS England recovery plan [11, 20].

## Results

**Fig. 2:**
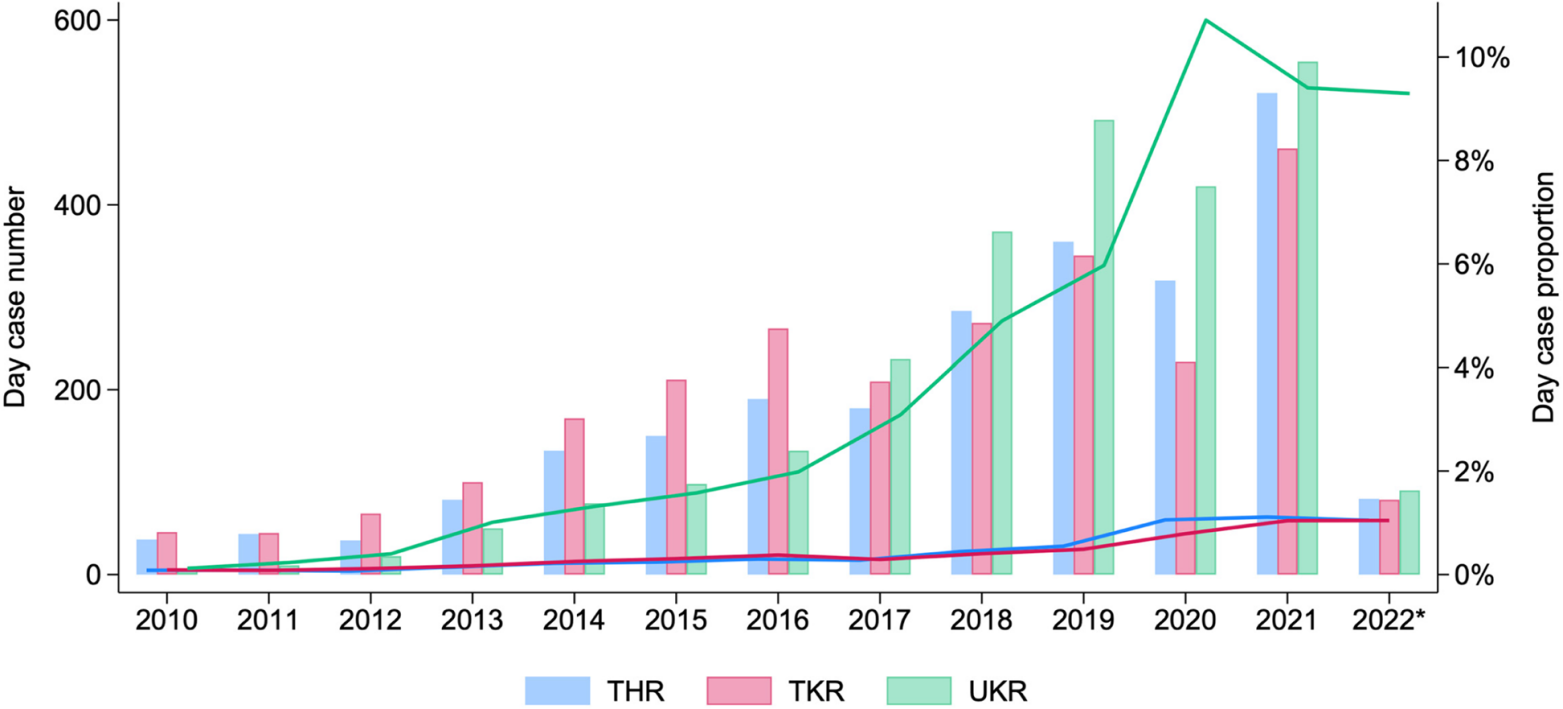
Day case procedure incidence by year, as a proportion of all activity, by type of joint replacement. Bars represent absolute numbers, lines represent proportions. *2022 data to March 31st. THR total hip replacement, TKR total knee replacement, UKR unicompartmental knee replacement

The study population comprised 68,232 procedures, of which 7485 were day case and 60,747 were 1-day-stay inpatient. Patient characteristics are summarised in Table 1, with additional details provided in Additional file 1: Figures S1 to S3. The incidence of day case surgery increased over time with 52.9% of cases occurring from 2019 onwards. Day case procedures represented 1.05% of all THRs and TKRs, and 9.3% of all UKRs in 2022 (Fig. 2). Across the study period, 287 of 407 units (70.5%) nationally performed day case procedures. Details of individual unit activity per year are provided in Additional file 1: Figures S10 to S12.

**Table 1:** Summary of patient characteristics. Numbers in brackets represent standard deviation for continuous variables, and percentages for categorical variables. Further details of all variables can be found in Additional file 1. *LOS* length of stay, *ASA* American Society of Anaesthesiologists, *IMD* Index of Multiple Deprivation, *NHS* National Health Service, *VTEP* venous thromboembolism prophylaxis

	Total hip replacement		Total knee replacement		Unicompartmental knee replacement		
	Day case	Inpatient (LOS 1)	Day case	Inpatient (LOS 1)	Day case	Inpatient (LOS 1)	
	(*N* = 2420)	(*N* = 29,307)	(*N* = 2509)	(*N* = 21,402)	(*N* = 2556)	(*N* = 2556)	
Age	63.90 (10.95)	63.74 (11.09)	66.66 (8.84)	66.92 (8.68)	63.54 (9.34)	62.80 (9.65)	
Sex							
Female	1203 (49.7%)	12,957 (44.2%)	1188 (47.3%)	8724 (40.8%)	1137 (44.5%)	3919 (39.0%)	
Male	1217 (50.3%)	16,350 (55.8%)	1321 (52.7%)	12,678 (59.2%)	1419 (55.5%)	6119 (61.0%)	
ASA grade							
I—Fit and healthy	456 (18.8%)	4723 (16.1%)	279 (11.1%)	1998 (9.3%)	461 (18.0%)	1,618 (16.1%)	
II—Mild disease not incapacitating	1802 (74.5%)	21,262 (72.5%)	2007 (80.0%)	16,532 (77.2%)	1898 (74.3%)	7528 (75.0%)	
III—Incapacitating systemic disease	161 (6.7%)	3288 (11.2%)	221 (8.8%)	2849 (13.3%)	197 (7.7%)	889 (8.9%)	
IV–V—Life threatening disease	1 (0.0%)	34 (0.1%)	2 (0.1%)	23 (0.1%)	0 (0.0%)	3 (0.0%)	
Charlson Comorbidity Index							
None	1491 (61.6%)	17,445 (59.5%)	1365 (54.4%)	11,233 (52.5%)	1495 (58.5%)	6038 (60.2%)	
Mild (1–2)	756 (31.2%)	9372 (32.0%)	918 (36.6%)	7972 (37.2%)	851 (33.3%)	3234 (32.2%)	
Moderate (3–4)	116 (4.8%)	1857 (6.3%)	175 (7.0%)	1678 (7.8%)	148 (5.8%)	595 (5.9%)	
Severe (5 +)	57 (2.4%)	633 (2.2%)	51 (2.0%)	519 (2.4%)	62 (2.4%)	171 (1.7%)	
No admissions in prior year	1667 (68.9%)	20,311 (69.3%)	1660 (66.2%)	14,186 (66.3%)	1764 (69.0%)	6650 (66.2%)	
Previous contralateral procedure	391 (16.2%)	5931 (20.2%)	483 (19.3%)	5474 (25.6%)	465 (18.2%)	1767 (17.6%)	
Obesity	838 (34.6%)	12,342 (42.1%)	1276 (50.9%)	12,196 (57.0%)	1312 (51.3%)	5086 (50.7%)	
Mental health diagnosis	595 (24.6%)	8446 (28.8%)	523 (20.8%)	5738 (26.8%)	729 (28.5%)	2728 (27.2%)	
Smoking	88 (3.6%)	1431 (4.9%)	71 (2.8%)	959 (4.5%)	98 (3.8%)	494 (4.9%)	
Ethnicity: white	2180 (98.2%)	27,056 (98.2%)	2,259 (95.6%)	20,072 (97.4%)	2341 (96.8%)	9344 (97.2%)	
Residence: urban	1603 (66.2%)	19,163 (65.4%)	1777 (70.8%)	14,449 (67.5%)	1754 (68.6%)	6772 (67.5%)	
IMD quantile							
Least deprived	540 (22.3%)	6096 (20.8%)	493 (19.6%)	4143 (19.4%)	764 (29.9%)	2625 (26.2%)	
2	600 (24.8%)	7131 (24.3%)	621 (24.8%)	5103 (23.8%)	610 (23.9%)	2530 (25.2%)	
3	555 (22.9%)	6786 (23.2%)	579 (23.1%)	4947 (23.1%)	545 (21.3%)	2243 (22.3%)	
4	426 (17.6%)	5289 (18.0%)	443 (17.7%)	4052 (18.9%)	375 (14.7%)	1562 (15.6%)	
Most deprived	288 (11.9%)	3768 (12.9%)	364 (14.5%)	3036 (14.2%)	260 (10.2%)	1048 (10.4%)	
Missing	11 (0.5%)	237 (0.8%)	9 (0.4%)	121 (0.6%)	2 (0.1%)	30 (0.3%)	
Unit sector							
NHS	1167 (48.2%)	19,824 (67.6%)	837 (33.4%)	13,499 (63.1%)	2,038 (79.7%)	6255 (62.3%)	
Independent	1253 (51.8%)	9483 (32.4%)	1672 (66.6%)	7903 (36.9%)	518 (20.3%)	3783 (37.7%)	
Anaesthetic: neuraxial only	2069 (85.5%)	23,573 (80.4%)	2087 (83.2%)	17,855 (83.4%)	1353 (52.9%)	6254 (62.3%)	
VTEP: chemical and mechanical	2014 (83.2%)	25,319 (86.4%)	2175 (86.7%)	18,485 (86.4%)	2303 (90.1%)	9263 (92.3%)	
Indication: OA only	2278 (94.1%)	27,051 (92.3%)	2456 (98.8%)	20,905 (99.1%)	2514 (98.5%)	9871 (98.5%)	

### Primary outcome

**Fig. 3:**
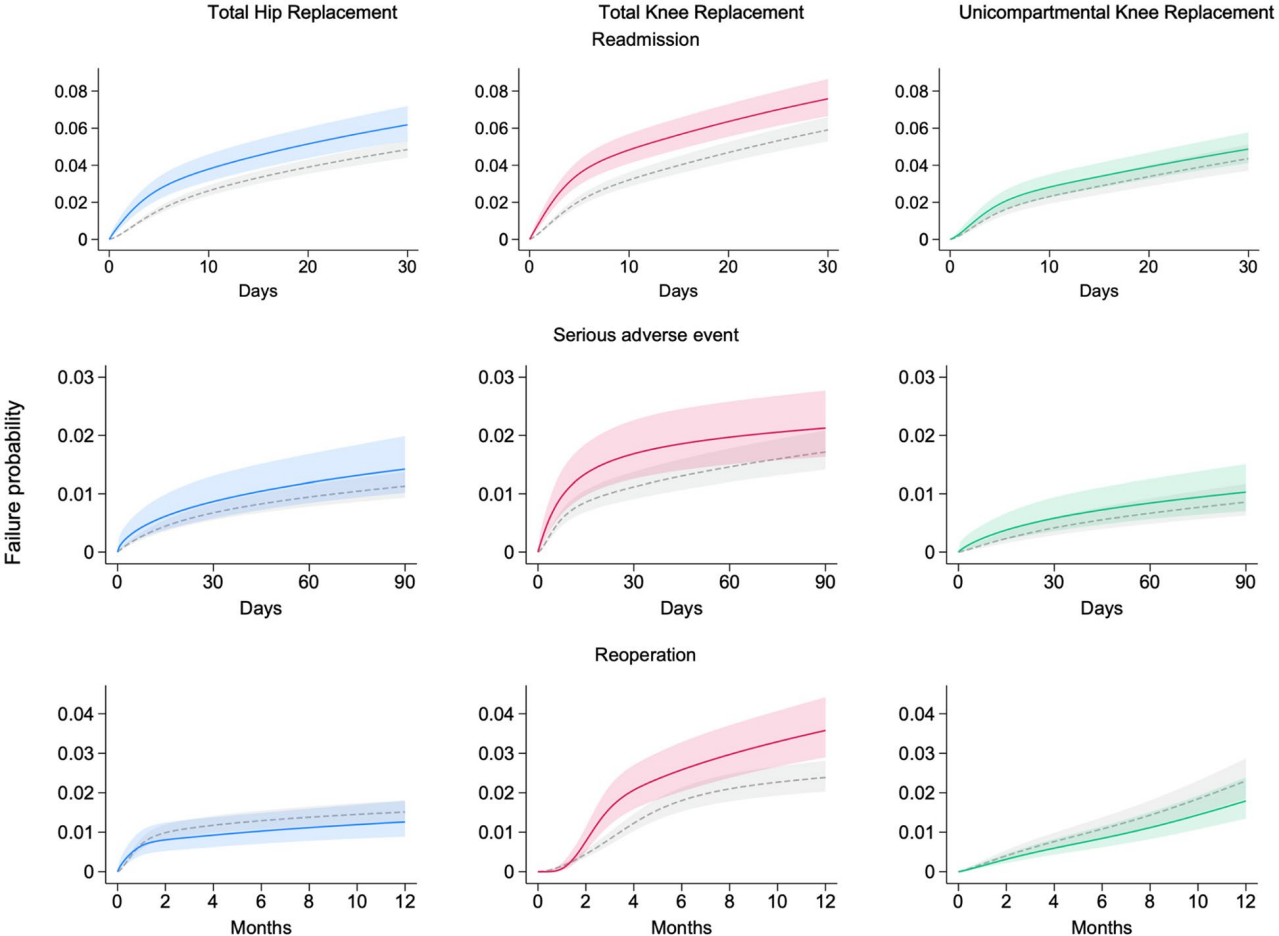
Adjusted probabilities for each outcome for day case compared to 1-day inpatient joint replacement, generated using standardised survival estimates from flexible parametric survival models. Coloured lines represent day cases, and grey dashed lines represent the inpatient comparison group. Blue = THR, red = TKR, green = UKR. Shaded areas represent 95% confidence intervals

Day case surgery was associated with significantly higher risk of 30-day readmission for THR (adjusted relative risk (RR) 1.28, 95% CI 1.07 to 1.53; absolute risk difference (RD) 1.33%, 95% CI 0.29 to 2.38; number needed to harm (NNTH) 75; Fig. 3), and TKR (RR 1.28, 95% CI 1.10 to 1.48; RD 1.67%, 95% CI 0.48 to 2.87; NNTH 60). There was no statistically significant difference for UKR (RR 1.12, 95% CI 0.88, 1.41). The risk of readmission significantly varied over follow-up time after joint replacement and was raised for day cases until day four following THR and day five following TKR (Additional file 1: Figure S13). The commonest reasons for readmission can be found in Additional file 1: Figure S14.

**Fig. 4:**
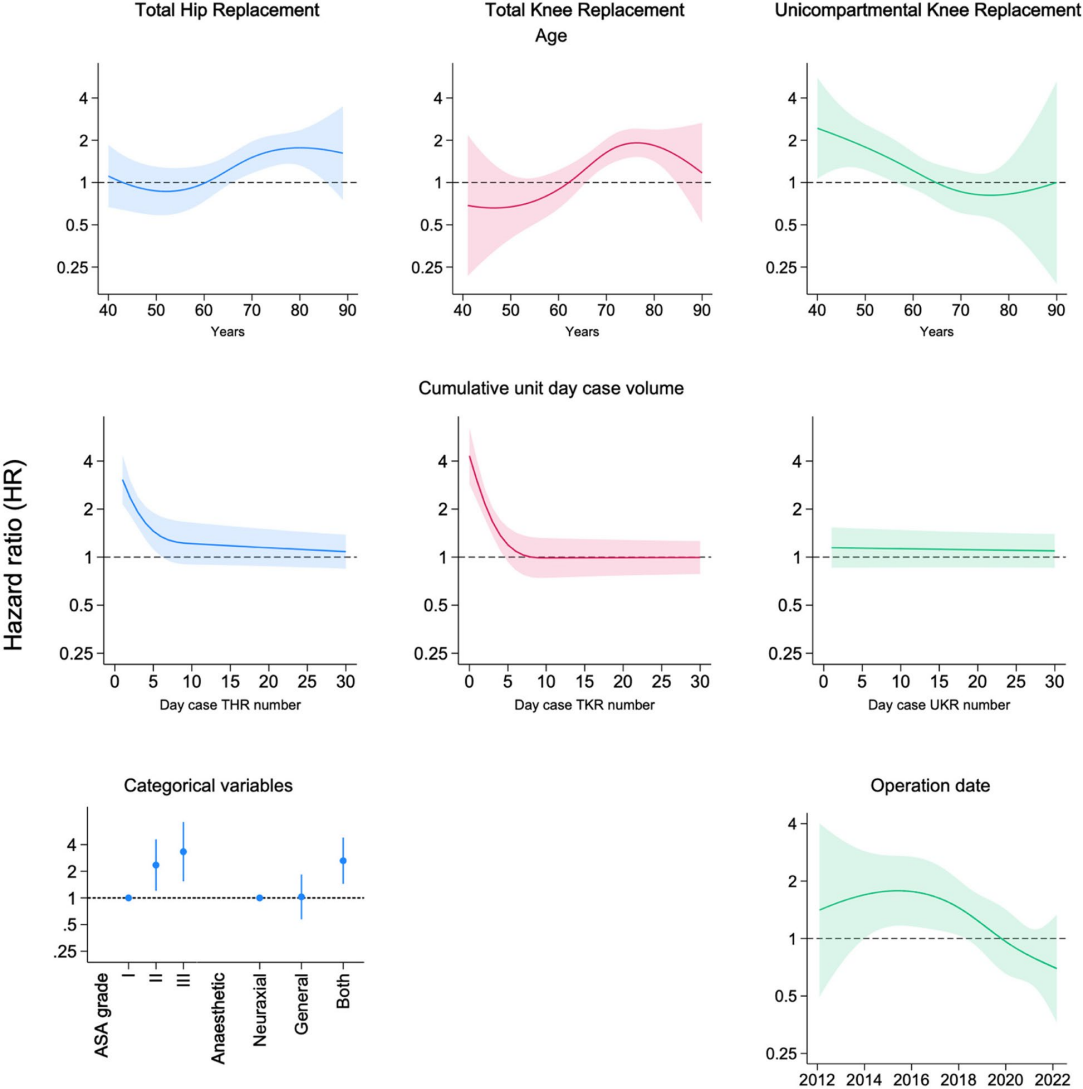
Risk factors for 30-day readmission specifically for day case joint replacement. These were modelled by including each variable as an interaction term, with significant results included in the final model and displayed here. General risk factors for both day case and inpatient procedures are displayed in the supplementary materials. The top row shows the association between age and risk of day case readmission. The middle row shows a significant hospital-level learning curve for day case THR and TKR, but not UKR. The bottom row demonstrates categorical variables associated with day case THR readmission, and a time effect for day case UKR. Blue = THR, red = TKR, green = UKR. Shaded areas for continuous variables and bars for categorical variables represent 95% confidence intervals

Day case specific factors associated with increased readmission included age of more than 66 years for THR and TKR, and age of less than 57 years for UKR (Fig. 4). There was a significant unit learning effect, where the first 6-day case THRs and the first 4-day case TKRs per unit were significantly more likely to be readmitted. After these unit volumes were surpassed, readmission risks became equivalent. The first day case THR per hospital was associated with a hazard ratio (HR) of 3.08 (95% CI 2.16, 4.38), and the first day case TKR per hospital 2.84 (95% CI 2.11, 3.84). There was no such learning effect for UKR, but instead a time effect where day case UKRs undertaken prior to 2018 were associated with significantly increased readmission risk (Fig. 4). Other day-case specific risk factors included an ASA grade of II or III, and a combination of general and neuraxial anaesthesia (compared to neuraxial alone) associated with increased readmission risks for THR (Fig. 4).

### Secondary outcomes

Day case surgery was not associated with any significant difference in risk of 90-day serious adverse events for any procedure in adjusted analysis (Fig. 3): THR RR 1.26 (95% CI 0.85, 1.85), TKR 1.24 (95% CI 0.89, 1.72), and UKR 1.20 (95% CI 0.74, 1.96). There was no significant difference with 90-day mortality as the endpoint specifically (Additional file 1: Figure S15). There was one significant risk factor for 90-day serious adverse events following day case surgery; a Charlson Comorbidity Index score of 1 or more was associated with increased risk for TKR (Additional file 1: Figure S16).

Day case TKR was associated with a significantly higher risk of reoperation at 1 year than inpatient TKR, RR 1.50 (95% CI 1.15, 1.96; RD 1.19%, 95% CI 0.34 to 2.04; NNTH 84; Fig. 3). The most common reoperation was manipulation under anaesthesia (MUA, 2.48% of day case TKRs; Table 2, Additional file 1: Figures S17 and S18). There were no significant differences in 1-year reoperation risks for THR (RR 0.83, 95% CI 0.56, 1.24) and UKR (RR 0.78, 95% CI 0.54, 1.12). For THR, day case procedures performed in the independent sector were associated with significantly lower 1-year reoperation risk (HR 0.36, 95% CI 0.15, 0.87), and day case THRs where a nerve block was performed were associated with a significantly higher risk (HR 9.95, 95% CI 2.49, 39.7; Additional file 1: Figure S16).

**Table 2:** Crude outcomes. *Not mutually exclusive. *LOS* length of stay, *MI* myocardial infarction, *VTE* venous thromboembolism, *UTI* urinary tract infection, *NJR* National Joint Registry, *HES* Hospital Episode Statistics, *PP fracture* peri-prosthetic fracture, *MUA* manipulation under anaesthesia

	Total hip replacemen				Total knee replacement				Unicompartmental knee replacement				
	Day Case		Inpatient (LOS=1)		Day Case		Inpatient (LOS=1)		Day Case		Inpatient (LOS=1)		
	*N* =	%	*N* =	%	*N* =	%	*N* =	%	*N* =	%	*N* =		%
Total	2420		29,307		2509		21,402		2556		10,038		
30-day readmissions	151	6.24	1362	4.65	189	7.53	1261	5.89	124	4.85	392		3.91
90-day serious adverse events*	34	1.4	341	1.16	53	2.12	352	1.64	26	1.02	87		0.87
MI	3	0.12	32	0.11	5	0.2	26	0.12	4	0.16	9		0.09
Stroke	3	0.12	19	0.06	6	0.24	15	0.07	0	0	6		0.06
VTE	12	0.5	102	0.35	18	0.72	149	0.70	10	0.39	30		0.30
Pneumonia	14	0.58	137	0.47	21	0.84	125	0.58	8	0.31	26		0.26
UTI	9	0.37	68	0.23	11	0.44	60	0.28	4	0.16	23		0.23
Death	2	0.08	28	0.10	4	0.16	33	0.15	2	0.08	4		0.04
1-year reoperations*	31	1.28	413	1.41	84	3.36	468	2.19	45	1.76	242		2.41
Revision (NJR)	17	0.7	218	0.74	14	0.56	70	0.33	14	0.55	74		0.74
Additional reoperations (HES)*:	14	0.58	195	0.67	70	2.8	398	1.86	31	1.21	168		1.67
Debridement/washout	3	0.12	42	0.14	7	0.28	50	0.23	12	0.47	59		0.59
PP fracture fixation/revision	1	0	11	0.04	0	0	11	0.05	0	0	8		0.08
Dislocation reduction	11	0.45	143	0.49	-	-	-	-	-	-	-		-
MUA	-	-	-	-	62	2.48	327	1.53	10	0.39	33		0.33
Other knee procedure	-	-	-	-	3	0.12	30	0.14	12	0.47	83		0.83
Above-knee amputation	-	-	-	-	0	0	2	0.01	0	0	0		0

Sensitivity analyses showed similar results when missing data were included in univariable models, and for alternative statistical analyses performed to account for data clustering within units and surgeons (Additional file 1: Figures S19–S21). Risk factors for each outcome for the entire study population, i.e. both day cases and inpatients rather than day cases specifically, are displayed in Additional file 1: Figures S22–S27. Long-term crude readmission and mortality rates were comparable between day case and 1-day stay inpatients, but not multiple-day stay inpatients, validating the 1-day inpatient comparison group (Additional file 1: Figure S28).

## Discussion

This study used routinely collected national registry data to compare outcomes of NHS-funded day-case hip and knee replacements in England with inpatient procedures involving a 1-night hospital stay. Day case THR and TKR were associated with increased risk of readmission, primarily within five days postoperatively, with NNTH of 75 and 60, respectively. A hospital-level learning curve was identified, where the first six day case THRs and the first four TKRs performed per hospital were associated with higher readmission risk. Despite this, there was no difference in the risk of serious adverse events. Day case TKR was however associated with a significantly higher risk of reoperation within one year (NNTH = 84). The majority of these reoperations were manipulations under anaesthesia (MUA), which are typically performed to address postoperative stiffness. No increased reoperation risk was observed for day-case THR, and no difference was observed in any of the outcomes for day case UKR.

This is the first study of its kind in the NHS setting, and the largest national cohort study of day case hip and knee replacement, with the longest follow-up period in the literature. Strengths include the use of nationally representative mandatory datasets, enabling reliable long-term prospective capture of adverse outcomes and providing generalisable, real-world evidence. Other strengths include the comparison group being one-day inpatients rather than all inpatients, reducing confounding prior to further statistical adjustment, and the application of modern epidemiological techniques in the analysis. Limitations include the observational nature of the study, where associations have been identified, but causation cannot be proven. Preoperative intention for day-case surgery was not recorded, limiting the findings to a per-protocol rather than intention-to-treat interpretation. Confounding variable accuracy is not routinely audited in NJR or HES. Patient-reported outcome measures (PROMs) were not analysed due to ongoing issues with national data availability [21]. National-level data did not permit detailed examination of specific components of local day-case pathways, despite unit-level effects being broadly accounted for through sensitivity analyses. Finally, any shift of the care burden onto emergency departments (not leading to admission), general practitioners, and informal carers was not assessed; however, a recent Danish study found no significant differences in healthcare system contacts [22].

A 2024 systematic review and meta-analysis reported comparable outcomes for day-case hip and knee replacement based on 38 comparative studies [12]. However, the evidence was judged to be mostly of very low quality, observational, single-centre studies, with high risk of bias, short follow-up periods (30 to 90 days), and significant residual confounding. There have been two small RCTs on day case THR, but neither were powered to detect differences in safety outcomes, with follow-up periods of less than three months [23, 24]. There is one other national cohort study in the literature, which used Danish national inpatient registry data and reported no difference in 30- or 90-day readmissions for 3498 day case compared to one-day stay inpatient THR, TKR, and UKR [25]. The difference to our readmission risk could be explained by day case joint replacement being more established in Denmark, with overall proportions of more than double those described here [26]; therefore, higher procedural volumes may mitigate the initial unit learning curve observed here as the practice becomes more established. Previous UK-specific comparative studies comprise two single-centre observational studies on day case UKR and THR, which reported no increased readmissions or adverse events, though patient numbers were below the NNTH identified in this study [27, 28].

The learning curve effect for the first four and six TKR and THRs done as day case per unit has not been investigated or reported elsewhere. Possible explanations include improvements in local pathways through experience, changes in patient advice regarding when to seek help, and shifts in staff attitudes which subsequently impact patient confidence. Alternatively, as day case intention was not recorded, it might reflect patients who were not planned for day case but were discharged in an opportunistic manner in units without formal day case pathways. Several previous reports highlight the impact of a unit’s culture, where significant reductions in general inpatient length of stay are seen with the establishment of day case pathways, even for procedures not in the pathway [27, 29]. Notably, the learning curve was confined to readmissions and did not lead to an increase in serious adverse medical events or reoperations, implicating the “softer” factors described above. Further evidence for the role of patient confidence is provided by the fact that previous contralateral surgery was a significant protective factor against readmission generally for THR and TKR (Supplementary Figs. 22 and 24). There was no such association for UKR, nor learning curve, despite a similar progression in active day case units (Supplementary Figs. 26 and 12). This may be because UKR is more suited to day case procedures, requiring less extensive dissection, causing less postoperative pain, and generally resulting in shorter lengths of stay than TKR [30, 31].

The novel finding of increased one-year reoperation risk for day-case TKR is concerning. Although an MUA can lead to improved range of motion, patients who have undergone it have poorer PROMs and higher risk of subsequent revision [32, 33]. There is one previous similar multicentre study with follow-up of more than 90 days; a registry study of 16,303 day case hip and knee replacement patients in Ontario, Canada, reporting equivalent or superior outcomes following day case compared to a propensity-matched subset of all inpatients up to 1 year [34]. However, TKR and UKR were grouped together, and non-revision reoperations such as MUA were not considered. Furthermore, the comparison group was all inpatients rather than one-day stay inpatients, decreasing validity due to fundamental differences between patients suitable for day case and those who require prolonged inpatient admission. These differences cannot be fully accounted for by propensity score matching, which, if used in this setting, exaggerates bias [35]. Other large studies were from the US, where generalisability to other non-insurance-based healthcare systems is doubtful. Of three larger US observational studies, two reported equivalent or superior 90-day outcomes for day cases [36, 37], and one reported significantly higher odds of 90-day complications (including some reoperations) for day case TKR [38].

Further work is urgently needed to explore the observed increase in reoperations after day-case TKR. One possible explanation is differences in physiotherapy provision. To our knowledge, no published NHS day case TKR protocol includes day one outpatient physiotherapy [11, 39, 40], and national guidelines recommend outpatient physiotherapy is not routinely offered after joint replacement [41]. Whereas for inpatients, national guidelines recommend a 7-day physiotherapy service to allow prompt discharge [11, 42]. Day case patients could be disadvantaged by the absence of physiotherapy on day one, when the effects of local anaesthetic and regional blocks have worn off and pain has increased, with a lack of movement due to reduced confidence leading to stiffness. This is supported by second-side primary TKR being a significant protective factor against reoperation in both groups (Supplementary Fig. 24). In-person physiotherapy following TKR is associated with significantly increased range of motion and decreased MUA rates [43], and intensive physiotherapy regimes are standard practice following an MUA [44].

Alternative explanations for the higher reoperation rate include other differences between inpatient and day case pathways such as analgesia, or residual confounding where day case patients are selected because they are fitter and more active and may therefore have a lower threshold to undergo MUA. Higher preoperative mobility, which was not directly controlled for in this study, has previously been associated with higher risk of MUA [45]. A large RCT, if feasible, is ultimately required to prove causation and identify best practices for day case protocols. In the meantime, prospective monitoring could be efficiently achieved by amending the NJR Minimum Data Set to include day case status on an intention to treat basis. Consideration should be given for physiotherapy input from day one following day case TKR; an MUA costs the NHS on average £4,317 [46], and a home physiotherapy visit £57.26 [47]. However, the provision of outpatient physiotherapy in England is variable, and it may be difficult to accommodate increased demand in an already stretched system [48]. There will be subsequent need for a health economic evaluation of the entire day case pathway incorporating both inpatient and community costs. Internationally, similar large studies using robust methodologies across different healthcare systems are warranted to examine whether the present findings are generalisable outside the NHS.

## Conclusions

This study was the first to evaluate NHS-funded day case hip and knee replacements on a national level addressing a key research priority amidst plans for rapid expansion of the practice. Day case THR and TKR were associated with a higher risk of readmission compared to 1-day stay inpatients, primarily within 5 days postoperatively, with a hospital-level learning curve observed during initial adoption. Other risk factors have been identified which may inform patient selection. Whilst no difference in serious adverse events was identified, day-case TKR was associated with an increased risk of reoperation within one year, predominantly MUA.

Day case UKR was not associated with any increase in adverse outcomes and appears safe. Day case THR appears mostly safe, provided the increased unplanned readmission risk in new centres is acceptable to stakeholders with its expansion. Further research is urgently required to address the increased risks observed with day case TKR. In the meantime, routine physiotherapy input from day one following day case TKR, which may mitigate the observed differences in outcomes, should be considered.

## Supplementary Information

Additional File 1: Figures S1–S26. Fig S1 – Flowchart showing HES data cleaning rules. Fig S2–S4 – Summary characteristics for THR, TKR, and UKR patients, including those with LOS > 1 day. Fig S5 – ICD-10 diagnosis codes used to define comorbidities and complications. Fig S6 – OPCS-4 procedure codes used to define reoperations. Fig S7 – Variables included in flexible parametric survival models. Fig S8–S9 – NJR data cleaning process flowcharts for hipand kneeprocedures. Fig S10–S12 – Proportion of units performing day case surgery by year for THR, TKR, and UKR. Fig S13 – Adjusted hazard ratios over time for day case vs inpatient outcomes, by procedure. Fig S14 – Most common primary ICD-10 diagnoses for 30-day readmissions, shown separately for day case and inpatient groups, by procedure. Fig S15 – Adjusted mortality probabilities for day case vs inpatient joint replacement, by procedure. Fig S16 – Risk factors for 90-day serious adverse events and one-year reoperations, by procedure. Fig S17 – Indications for revision surgery recorded in NJR, by procedure. Fig S18 – Most common OPCS-4 codes for non-revision reoperations within one year, by procedure. Fig S19–S21 – Flexible parametric survival model estimates for THR, TKR, and UKR. Fig S22, S24, S26 – Categorical risk factors for outcomes following THR, TKR, and UKR. Fig S23, S25 – Continuous risk factors for outcomes following THR, TKR, and UKR. Fig S28 – Long-term outcomes stratified by length of stay.

## Data Availability

The datasets generated and analysed in the current study are not publicly available due to data protection regulations. Access to data is limited to the researchers who have obtained permission for data processing through the NJR Research Committee. Further inquiries can be made to the corresponding author.

## Abbreviations

AIC: Akaike Information Criterion
ASA: American Society of Anaesthesiologists
BIC: Bayesian Information Criterion
BMI: Body mass index
CI: Confidence interval
CJRR: Canadian Joint Replacement Registry
DVT: Deep vein thrombosis
GIRFT: Getting It Right First Time
HES: Hospital Episode Statistics
HR: Hazard ratio
HVLC: High volume low complexity
ICD-10: International Classification of Diseases, 10th Revision
IMD: Index of Multiple Deprivation
LOS: Length of stay
MI: Myocardial infarction
MUA: Manipulation under anaesthesia
NHS: National Health Service
NICE: National Institute for Health and Care Excellence
NIHR: National Institute for Health Research
NJR: National Joint Registry
NNTH: Number needed to harm
OA: Osteoarthritis
OPCS: Classification of Interventions and Procedures, 4th Revision
ORUK: Orthopaedic Research UK
PE: Pulmonary embolism
PROMs: Patient Reported Outcome Measures
RCT: Randomised controlled trial
RD: Risk difference
RR: Relative risk
THR: Total hip replacement
TKR: Total knee replacement
UKR: Unicompartmental knee replacement
UTI: Urinary tract infection
VTE: Venous thromboembolism
