# Supplementary material for: Day case hip and knee replacement in England: a population-based cohort study using linked National Joint Registry and Hospital Episode Statistics data
Source: BMC Med. 2025 Oct 14;23:564. doi: 10.1186/s12916-025-04280-y (PMC12523212; doi:10.1186/s12916-025-04280-y)
Supplement: Supplementary file 1 — Additional File 1: Figures S1–S26. Fig S1 – Flowchart showing HES data cleaning rules. Fig S2–S4 – Summary characteristics for THR, TKR, and UKR patients, including those with LOS > 1 day. Fig S5 – ICD-10 diagnosis codes used to define comorbidities and complications. Fig S6 – OPCS-4 procedure codes used to define reoperations. Fig S7 – Variables included in flexible parametric survival models. Fig S8–S9 – NJR data cleaning process flowcharts for hipand kneeprocedures. Fig S10–S12 – Proportion of units performing day case surgery by year for THR, TKR, and UKR. Fig S13 – Adjusted hazard ratios over time for day case vs inpatient outcomes, by procedure. Fig S14 – Most common primary ICD-10 diagnoses for 30-day readmissions, shown separately for day case and inpatient groups, by procedure. Fig S15 – Adjusted mortality probabilities for day case vs inpatient joint replacement, by procedure. Fig S16 – Risk factors for 90-day serious adverse events and one-year reoperations, by procedure. Fig S17 – Indications for revision surgery recorded in NJR, by procedure. Fig S18 – Most common OPCS-4 codes for non-revision reoperations within one year, by procedure. Fig S19–S21 – Flexible parametric survival model estimates for THR, TKR, and UKR. Fig S22, S24, S26 – Categorical risk factors for outcomes following THR, TKR, and UKR. Fig S23, S25 – Continuous risk factors for outcomes following THR, TKR, and UKR. Fig S28 – Long-term outcomes stratified by length of stay. [file 12916_2025_4280_MOESM1_ESM.docx]

Supplementary materials


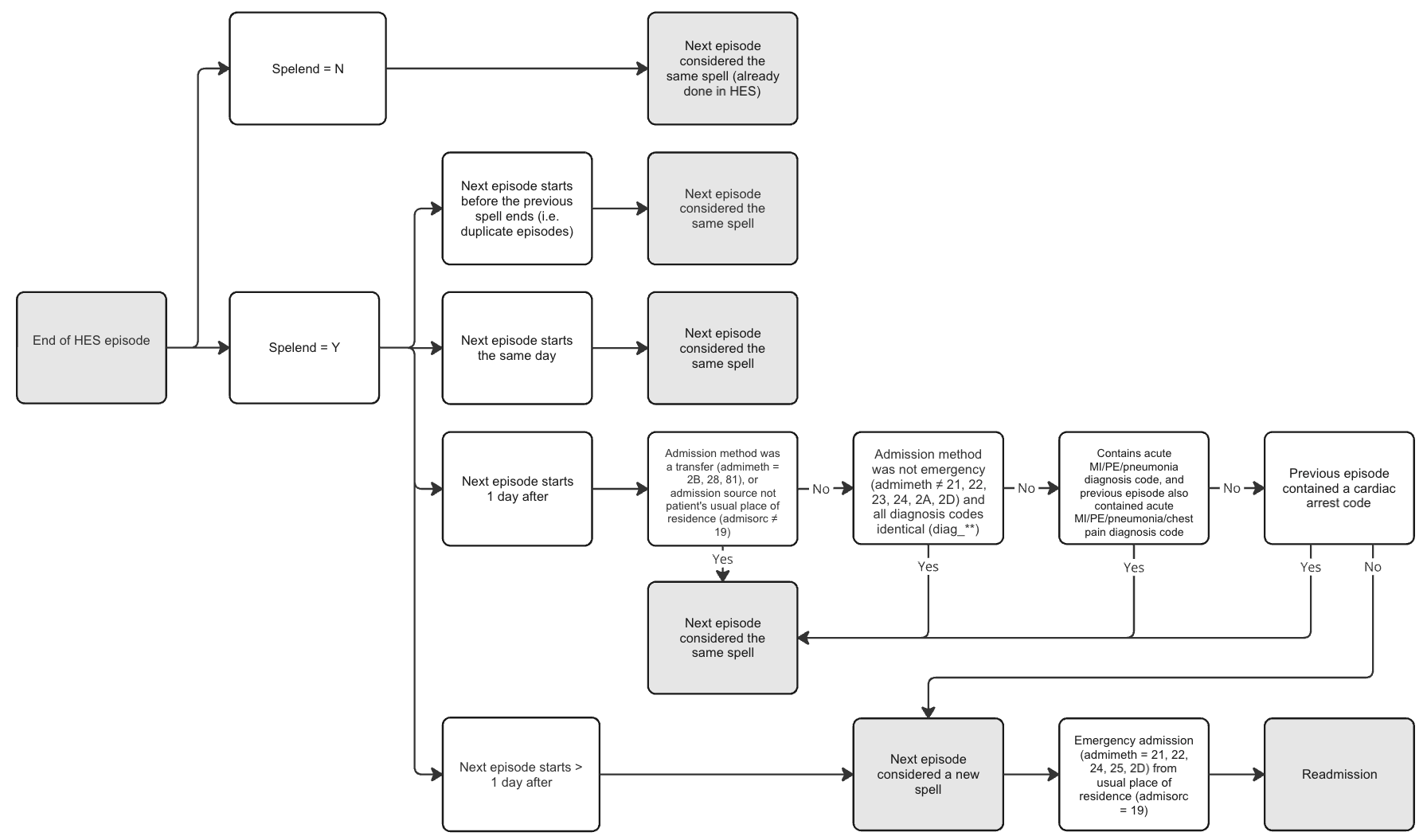


Figure S1 - Flowchart showing HES data cleaning rules.

| Total Hip Replacement | | | |
| --- | --- | --- | --- |
|  | Day Case | Inpatient (LOS 1) | Inpatient (LOS 2+) |
|  | (N=2,420) | (N=29,307) | (N=636,888) |
| Age | 63.90 (10.95) | 63.74 (11.09) | 68.57 (11.22) |
| Sex |  |  |  |
| Female | 1,203 (49.7%) | 12,957 (44.2%) | 384,713 (60.4%) |
| Male | 1,217 (50.3%) | 16,350 (55.8%) | 252,175 (39.6%) |
| ASA grade |  |  |  |
| I - Fit and healthy | 456 (18.8%) | 4,723 (16.1%) | 75,440 (11.8%) |
| II - Mild disease not incapacitating | 1,802 (74.5%) | 21,262 (72.5%) | 439,890 (69.1%) |
| III - Incapacitating systemic disease | 161 (6.7%) | 3,288 (11.2%) | 118,307 (18.6%) |
| IV-V - Life threatening disease | 1 (0.0%) | 34 (0.1%) | 3,251 (0.5%) |
| Charlson Comorbidity Index |  |  |  |
| None | 1,491 (61.6%) | 17,445 (59.5%) | 338,185 (53.1%) |
| Mild (1-2) | 756 (31.2%) | 9,372 (32.0%) | 220,652 (34.6%) |
| Moderate (3-4) | 116 (4.8%) | 1,857 (6.3%) | 55,826 (8.8%) |
| Severe (5+) | 57 (2.4%) | 633 (2.2%) | 22,225 (3.5%) |
| Admissions in prior year |  |  |  |
| 0 | 1,667 (68.9%) | 20,311 (69.3%) | 383,527 (60.2%) |
| 1 | 509 (21.0%) | 6,330 (21.6%) | 161,624 (25.4%) |
| 2+ | 244 (10.1%) | 2,666 (9.1%) | 91,737 (14.4%) |
| Previous contralateral procedure |  |  |  |
| No | 2,029 (83.8%) | 23,376 (79.8%) | 557,704 (87.6%) |
| Yes | 391 (16.2%) | 5,931 (20.2%) | 79,184 (12.4%) |
| Obesity | 838 (34.6%) | 12,342 (42.1%) | 240,302 (37.7%) |
| Gastrointestinal disease | 1,073 (44.3%) | 13,550 (46.2%) | 301,827 (47.4%) |
| Respiratory disease | 542 (22.4%) | 7,102 (24.2%) | 166,705 (26.2%) |
| Circulatory disease | 1,182 (48.8%) | 15,508 (52.9%) | 401,478 (63.0%) |
| Diabetes | 246 (10.2%) | 2,879 (9.8%) | 94,032 (14.8%) |
| Neurological disease | 346 (14.3%) | 4,586 (15.6%) | 103,093 (16.2%) |
| Urinary tract disease | 666 (27.5%) | 8,401 (28.7%) | 216,483 (34.0%) |
| Mental health diagnosis | 595 (24.6%) | 8,446 (28.8%) | 139,151 (21.8%) |
| Smoking | 88 (3.6%) | 1,431 (4.9%) | 31,694 (5.0%) |
| Ethnicity: white | 2,180 (98.2%) | 27,056 (98.2%) | 605,481 (98.0%) |
| Residence |  |  |  |
| Urban | 1,603 (66.2%) | 19,163 (65.4%) | 454,115 (71.3%) |
| Rural | 815 (33.7%) | 10,101 (34.5%) | 181,256 (28.5%) |
| Unknown | 2 (0.1%) | 43 (0.1%) | 1,517 (0.2%) |
| IMD quantile |  |  |  |
| Least deprived | 540 (22.3%) | 6,096 (20.8%) | 153,027 (24.0%) |
| 2 | 600 (24.8%) | 7,131 (24.3%) | 155,764 (24.5%) |
| 3 | 555 (22.9%) | 6,786 (23.2%) | 139,963 (22.0%) |
| 4 | 426 (17.6%) | 5,289 (18.0%) | 106,620 (16.7%) |
| Most deprived | 288 (11.9%) | 3,768 (12.9%) | 76,166 (12.0%) |
| Missing | 11 (0.5%) | 237 (0.8%) | 5,348 (0.8%) |
| Unit Sector |  |  |  |
| NHS | 1,167 (48.2%) | 19,824 (67.6%) | 449,883 (70.6%) |
| Independent | 1,253 (51.8%) | 9,483 (32.4%) | 187,005 (29.4%) |
| Anaesthetic |  |  |  |
| GA | 225 (9.3%) | 3,184 (10.9%) | 135,692 (21.3%) |
| GA and neuraxial | 124 (5.1%) | 2,449 (8.4%) | 78,954 (12.4%) |
| Neuraxial | 2,069 (85.5%) | 23,573 (80.4%) | 418,799 (65.8%) |
| Nerve block only | 2 (0.1%) | 101 (0.3%) | 3,365 (0.5%) |
| VTE Prophylaxis |  |  |  |
| None | 1 (0.0%) | 16 (0.1%) | 337 (0.1%) |
| Chemical only | 360 (14.9%) | 3,241 (11.1%) | 25,872 (4.1%) |
| Mechanical only | 45 (1.9%) | 731 (2.5%) | 36,215 (5.7%) |
| Chemical and mechanical | 2,014 (83.2%) | 25,319 (86.4%) | 574,464 (90.2%) |
| Indication |  |  |  |
| Osteoarthritis only | 2,278 (94.1%) | 27,051 (92.3%) | 587,765 (92.3%) |
| Osteoarthritis plus other | 65 (2.7%) | 974 (3.3%) | 19,224 (3.0%) |
| Other indications only | 77 (3.2%) | 1,282 (4.4%) | 29,899 (4.7%) |
| Procedure |  |  |  |
| Total Hip Replacement | 2,412 (99.7%) | 28,985 (98.9%) | 631,740 (99.2%) |
| Hip Resurfacing | 8 (0.3%) | 322 (1.1%) | 5,148 (0.8%) |
| Surgical approach |  |  |  |
| Posterior | 1,695 (70.0%) | 23,433 (80.0%) | 435,312 (68.3%) |
| Hardinge/Anterolateral/Lateral | 588 (24.3%) | 4,407 (15.0%) | 176,533 (27.7%) |
| Anterior | 72 (3.0%) | 473 (1.6%) | 978 (0.2%) |
| Other | 65 (2.7%) | 994 (3.4%) | 24,065 (3.8%) |
| Bearing |  |  |  |
| MoP | 1,170 (48.3%) | 15,486 (52.8%) | 375,778 (59.0%) |
| CoP | 848 (35.0%) | 10,699 (36.5%) | 163,463 (25.7%) |
| CoC | 364 (15.0%) | 2,445 (8.3%) | 75,855 (11.9%) |
| Other/unclassified | 38 (1.6%) | 677 (2.3%) | 21,792 (3.4%) |
| Mean femoral head size (mm) | 31.95 (3.19) | 32.48 (3.45) | 31.73 (3.71) |

Figure S2 - Full summary characteristics of THR patients including those with a length of stay of more than one night (excluded from main analysis). LOS = Length of Stay; ASA = American Society of Anaesthesiologists; IMD = Index of Multiple Deprivation; MoP = Metal-on-polyethylene; CoP = Ceramic-on-polyethylene; CoC = Ceramic-on-ceramic; MoM = Metal-on-metal; CoM = Ceramic-on-metal; GA = General Anaesthetic; VTE = Venous Thromboembolism.

| Total Knee Replacement | | | |
| --- | --- | --- | --- |
|  | Day Case | Inpatient (LOS 1) | Inpatient (LOS 2+) |
|  | (N=2,509) | (N=21,402) | (N=692,027) |
| Age | 66.66 (8.84) | 66.92 (8.68) | 69.75 (9.23) |
| Sex |  |  |  |
| Female | 1,188 (47.3%) | 8,724 (40.8%) | 399,957 (57.8%) |
| Male | 1,321 (52.7%) | 12,678 (59.2%) | 292,070 (42.2%) |
| ASA grade |  |  |  |
| I - Fit and healthy | 279 (11.1%) | 1,998 (9.3%) | 52,263 (7.6%) |
| II - Mild disease not incapacitating | 2,007 (80.0%) | 16,532 (77.2%) | 503,655 (72.8%) |
| III - Incapacitating systemic disease | 221 (8.8%) | 2,849 (13.3%) | 133,847 (19.3%) |
| IV-V - Life threatening disease | 2 (0.1%) | 23 (0.1%) | 2,262 (0.3%) |
| Charlson Comorbidity Index |  |  |  |
| None | 1,365 (54.4%) | 11,233 (52.5%) | 331,009 (47.8%) |
| Mild (1-2) | 918 (36.6%) | 7,972 (37.2%) | 271,442 (39.2%) |
| Moderate (3-4) | 175 (7.0%) | 1,678 (7.8%) | 66,694 (9.6%) |
| Severe (5+) | 51 (2.0%) | 519 (2.4%) | 22,882 (3.3%) |
| Admissions in prior year |  |  |  |
| 0 | 1,660 (66.2%) | 14,186 (66.3%) | 408,730 (59.1%) |
| 1 | 590 (23.5%) | 5,133 (24.0%) | 184,104 (26.6%) |
| 2+ | 259 (10.3%) | 2,083 (9.7%) | 99,193 (14.3%) |
| Previous contralateral procedure |  |  |  |
| No | 2,026 (80.7%) | 15,928 (74.4%) | 580,484 (83.9%) |
| Yes | 483 (19.3%) | 5,474 (25.6%) | 111,543 (16.1%) |
| Obesity | 1,276 (50.9%) | 12,196 (57.0%) | 361,282 (52.2%) |
| Gastrointestinal disease | 1,258 (50.1%) | 11,440 (53.5%) | 361,039 (52.2%) |
| Respiratory disease | 620 (24.7%) | 5,694 (26.6%) | 196,185 (28.3%) |
| Circulatory disease | 1,530 (61.0%) | 13,886 (64.9%) | 490,238 (70.8%) |
| Diabetes | 394 (15.7%) | 3,019 (14.1%) | 140,855 (20.4%) |
| Neurological disease | 489 (19.5%) | 4,207 (19.7%) | 129,873 (18.8%) |
| Urinary tract disease | 792 (31.6%) | 7,116 (33.2%) | 254,226 (36.7%) |
| Mental health diagnosis | 523 (20.8%) | 5,738 (26.8%) | 138,851 (20.1%) |
| Smoking | 71 (2.8%) | 959 (4.5%) | 31,116 (4.5%) |
| Ethnicity: white | 2,259 (95.6%) | 20,072 (97.4%) | 633,322 (93.4%) |
| Residence |  |  |  |
| Urban | 1,777 (70.8%) | 14,449 (67.5%) | 516,847 (74.7%) |
| Rural | 729 (29.1%) | 6,924 (32.4%) | 173,624 (25.1%) |
| Unknown | 3 (0.1%) | 29 (0.1%) | 1,556 (0.2%) |
| IMD quantile |  |  |  |
| Least deprived | 493 (19.6%) | 4,143 (19.4%) | 150,033 (21.7%) |
| 2 | 621 (24.8%) | 5,103 (23.8%) | 159,954 (23.1%) |
| 3 | 579 (23.1%) | 4,947 (23.1%) | 151,466 (21.9%) |
| 4 | 443 (17.7%) | 4,052 (18.9%) | 125,423 (18.1%) |
| Most deprived | 364 (14.5%) | 3,036 (14.2%) | 100,683 (14.5%) |
| Missing | 9 (0.4%) | 121 (0.6%) | 4,468 (0.6%) |
| Unit Sector |  |  |  |
| NHS | 837 (33.4%) | 13,499 (63.1%) | 485,567 (70.2%) |
| Independent | 1,672 (66.6%) | 7,903 (36.9%) | 206,460 (29.8%) |
| Anaesthetic |  |  |  |
| GA | 302 (12.0%) | 2,627 (12.3%) | 151,097 (21.8%) |
| GA and neuraxial | 103 (4.1%) | 809 (3.8%) | 55,886 (8.1%) |
| Neuraxial | 2,087 (83.2%) | 17,855 (83.4%) | 479,335 (69.3%) |
| Nerve block only | 17 (0.7%) | 111 (0.5%) | 5,633 (0.8%) |
| VTE Prophylaxis |  |  |  |
| None | 1 (0.0%) | 12 (0.1%) | 495 (0.1%) |
| Chemical only | 283 (11.3%) | 2,521 (11.8%) | 26,965 (3.9%) |
| Mechanical only | 46 (1.8%) | 329 (1.5%) | 39,500 (5.7%) |
| Chemical and mechanical | 2,175 (86.7%) | 18,485 (86.4%) | 624,703 (90.3%) |
| Unknown | 4 (0.2%) | 55 (0.3%) | 364 (0.1%) |
| Indication |  |  |  |
| Osteoarthritis only | 2,456 (98.8%) | 20,905 (99.1%) | 670,384 (98.0%) |
| Osteoarthritis plus other | 15 (0.6%) | 83 (0.4%) | 4,599 (0.7%) |
| Other indications only | 14 (0.6%) | 103 (0.5%) | 9,057 (1.3%) |
| Approach |  |  |  |
| Medial parapatellar | 2,263 (90.3%) | 19,694 (92.3%) | 652,200 (94.3%) |
| Mid-Vastus | 63 (2.5%) | 1,161 (5.4%) | 19,514 (2.8%) |
| Sub-Vastus | 143 (5.7%) | 115 (0.5%) | 5,949 (0.9%) |
| Other | 36 (1.4%) | 377 (1.8%) | 13,999 (2.0%) |
| Bearing design |  |  |  |
| Unconstrained Fixed | 2,054 (81.9%) | 17,498 (81.8%) | 495,653 (71.6%) |
| Posterior Stabilised Fixed | 283 (11.3%) | 3,078 (14.4%) | 149,941 (21.7%) |
| Unconstrained Mobile | 51 (2.0%) | 373 (1.7%) | 26,278 (3.8%) |
| Posterior Stabilised Mobile | 30 (1.2%) | 54 (0.3%) | 4,912 (0.7%) |
| Constrained Condylar | 80 (3.2%) | 291 (1.4%) | 8,791 (1.3%) |
| Unclassified | 11 (0.4%) | 108 (0.5%) | 6,452 (0.9%) |

Figure S3 - Full summary characteristics of TKR patients including those with a length of stay of more than one night (excluded from main analysis). LOS = Length of Stay; ASA = American Society of Anaesthesiologists; IMD = Index of Multiple Deprivation; GA = General Anaesthetic; VTE = Venous Thromboembolism.

| Unicompartmental Knee Replacement | | | |
| --- | --- | --- | --- |
|  | Day Case | Inpatient (LOS 1) | Inpatient (LOS 2+) |
|  | (N=2,556) | (N=10,038) | (N=58,695) |
| Age | 63.54 (9.34) | 62.80 (9.65) | 63.40 (10.31) |
| Sex |  |  |  |
| Female | 1,137 (44.5%) | 3,919 (39.0%) | 30,097 (51.3%) |
| Male | 1,419 (55.5%) | 6,119 (61.0%) | 28,598 (48.7%) |
| ASA grade |  |  |  |
| I - Fit and healthy | 461 (18.0%) | 1,618 (16.1%) | 9,724 (16.6%) |
| II - Mild disease not incapacitating | 1,898 (74.3%) | 7,528 (75.0%) | 42,652 (72.7%) |
| III - Incapacitating systemic disease | 197 (7.7%) | 889 (8.9%) | 6,251 (10.6%) |
| IV-V - Life threatening disease | 0 (0.0%) | 3 (0.0%) | 68 (0.1%) |
| Charlson Comorbidity Index |  |  |  |
| None | 1,495 (58.5%) | 6,038 (60.2%) | 33,506 (57.1%) |
| Mild (1-2) | 851 (33.3%) | 3,234 (32.2%) | 20,186 (34.4%) |
| Moderate (3-4) | 148 (5.8%) | 595 (5.9%) | 3,845 (6.6%) |
| Severe (5+) | 62 (2.4%) | 171 (1.7%) | 1,158 (2.0%) |
| Admissions in prior year |  |  |  |
| 0 | 1,764 (69.0%) | 6,650 (66.2%) | 35,061 (59.7%) |
| 1 | 560 (21.9%) | 2,495 (24.9%) | 16,268 (27.7%) |
| 2+ | 232 (9.1%) | 893 (8.9%) | 7,366 (12.5%) |
| Previous contralateral procedure |  |  |  |
| No | 2,091 (81.8%) | 8,271 (82.4%) | 51,373 (87.5%) |
| Yes | 465 (18.2%) | 1,767 (17.6%) | 7,322 (12.5%) |
| Obesity | 1,312 (51.3%) | 5,086 (50.7%) | 28,873 (49.2%) |
| Gastrointestinal disease | 1,372 (53.7%) | 5,007 (49.9%) | 28,816 (49.1%) |
| Respiratory disease | 696 (27.2%) | 2,657 (26.5%) | 15,944 (27.2%) |
| Circulatory disease | 1,417 (55.4%) | 5,525 (55.0%) | 33,561 (57.2%) |
| Diabetes | 290 (11.3%) | 1,132 (11.3%) | 9,264 (15.8%) |
| Neurological disease | 505 (19.8%) | 1,746 (17.4%) | 10,494 (17.9%) |
| Urinary tract disease | 847 (33.1%) | 3,173 (31.6%) | 19,949 (34.0%) |
| Mental health diagnosis | 729 (28.5%) | 2,728 (27.2%) | 13,668 (23.3%) |
| Smoking | 98 (3.8%) | 494 (4.9%) | 3,130 (5.3%) |
| Ethnicity: white | 2,341 (96.8%) | 9,344 (97.2%) | 54,705 (95.4%) |
| Residence |  |  |  |
| Urban | 1,754 (68.6%) | 6,772 (67.5%) | 41,778 (71.2%) |
| Rural | 800 (31.3%) | 3,250 (32.4%) | 16,720 (28.5%) |
| Unknown | 2 (0.1%) | 16 (0.2%) | 197 (0.3%) |
| IMD quantile |  |  |  |
| Least deprived | 764 (29.9%) | 2,625 (26.2%) | 15,483 (26.4%) |
| 2 | 610 (23.9%) | 2,530 (25.2%) | 13,945 (23.8%) |
| 3 | 545 (21.3%) | 2,243 (22.3%) | 12,598 (21.5%) |
| 4 | 375 (14.7%) | 1,562 (15.6%) | 9,545 (16.3%) |
| Most deprived | 260 (10.2%) | 1,048 (10.4%) | 6,697 (11.4%) |
| Missing | 2 (0.1%) | 30 (0.3%) | 427 (0.7%) |
| Unit Sector |  |  |  |
| NHS | 2,038 (79.7%) | 6,255 (62.3%) | 38,455 (65.5%) |
| Independent | 518 (20.3%) | 3,783 (37.7%) | 20,240 (34.5%) |
| Anaesthetic |  |  |  |
| GA | 1,079 (42.2%) | 3,138 (31.3%) | 18,044 (30.7%) |
| GA and neuraxial | 94 (3.7%) | 548 (5.5%) | 4,331 (7.4%) |
| Neuraxial | 1,353 (52.9%) | 6,254 (62.3%) | 35,675 (60.8%) |
| Nerve block only | 30 (1.2%) | 98 (1.0%) | 636 (1.1%) |
| VTE Prophylaxis |  |  |  |
| None | 3 (0.1%) | 5 (0.0%) | 49 (0.1%) |
| Chemical only | 225 (8.8%) | 527 (5.3%) | 1,753 (3.0%) |
| Mechanical only | 17 (0.7%) | 224 (2.2%) | 3,348 (5.7%) |
| Chemical and mechanical | 2,303 (90.1%) | 9,263 (92.3%) | 53,513 (91.2%) |
| Unknown | 8 (0.3%) | 19 (0.2%) | 32 (0.1%) |
| Indication |  |  |  |
| Osteoarthritis only | 2,514 (98.5%) | 9,871 (98.5%) | 57,661 (98.4%) |
| Osteoarthritis plus other | 11 (0.4%) | 65 (0.6%) | 327 (0.6%) |
| Other indications only | 28 (1.1%) | 86 (0.9%) | 627 (1.1%) |
| Compartment |  |  |  |
| Medial | 2,286 (89.4%) | 8,547 (85.1%) | 42,803 (72.9%) |
| Lateral | 99 (3.9%) | 266 (2.6%) | 1,106 (1.9%) |
| Patello-femoral | 69 (2.7%) | 602 (6.0%) | 6,258 (10.7%) |
| Unspecified | 102 (4.0%) | 623 (6.2%) | 8,528 (14.5%) |
| Approach |  |  |  |
| Medial parapatellar | 2,193 (86.1%) | 8,810 (87.9%) | 52,626 (89.7%) |
| Mid-Vastus | 211 (8.3%) | 600 (6.0%) | 2,460 (4.2%) |
| Sub-Vastus | 32 (1.3%) | 227 (2.3%) | 951 (1.6%) |
| Other | 112 (4.4%) | 382 (3.8%) | 2,626 (4.5%) |
| Bearing design |  |  |  |
| Mobile | 1,406 (55.0%) | 4,341 (43.2%) | 30,486 (51.9%) |
| Fixed | 1,076 (42.1%) | 5,030 (50.1%) | 21,456 (36.6%) |
| Unclassified | 74 (2.9%) | 667 (6.6%) | 6,753 (11.5%) |

Figure S4 - Full summary characteristics of UKR patients including those with a length of stay of more than one night (excluded from main analysis). LOS = Length of Stay; ASA = American Society of Anaesthesiologists; IMD = Index of Multiple Deprivation; GA = General Anaesthetic; VTE = Venous Thromboembolism.

| **Diagnosis** | **ICD-10 codes** |
| --- | --- |
|  |  |
| *Comorbidities* |  |
| Charlson Comorbidity Index | See Quan et al, Coding algorithms for defining comorbidities in ICD-9-CM and ICD-10 administrative data. *Med Care* 2005; **43**(11): 1130-9. |
| Obesity | E66 |
| Gastrointestinal disease | K00-K93 |
| Respiratory disease | J00-J99 |
| Circulatory disease | I00-I99 |
| Diabetes | E100-149 |
| Neurological disease | G00-G99 |
| Urinary tract disease | N00-N99 |
| Mental health diagnosis | F00-F99 |
| Smoking | Z720 |
|  |  |
| *Serious adverse events* |  |
| Venous thromboembolism (VTE) | I26 I801-803 |
| Acute myocardial infarction (MI) | I21 I22 |
| Stroke | I60 I61 I63 I64 |
| Pneumonia | J12 J13 J14 J15 J18 J22 J440 J441 J69 J851 |
| UTI | N10 N300 N308 N309 N390 |
|  |  |
| *Peri-prosthetic fracture* |  |
| Hip | M966 M970 M971 M97 M8445 M8005 M8085 M8095 M8435 M8455 M846 M8475 M9075 S72 |
| Knee | M966 M971 M979 M8005 M8006 M8085 M8086 M8096 M8435 M8436 M8455 M8456 M8465 M8466 M9075 M9076 S72 S82 |
|  |  |

Figure S5 - ICD-10 diagnosis codes.

| **Diagnosis** | **OPCD-4 codes** |
| --- | --- |
| Reoperation | Laterality code matches NJR side + joint-specific procedure code |
|  | Laterality code matches NJR side + location code + non-joint specific procedure code |
| Reoperation for PP fracture | Periprosthetic fracture ICD-10 code + laterality code matches NJR side + joint-specific ORIF code OR location code + non-joint specific ORIF code |
|  |  |
| Laterality | Z943 Z942 Z941 |
|  |  |
| *Hip* |  |
| Location | Z843 Z902 Z761 Z762 Z763 Z756 W46 W47 W48 |
| *Joint-specific procedure codes* |  |
| Revision THR | W370 W372 W373 W374 W380 W382 W383 W384 W390 W392 W393 W394 W395 W472 W473 W483 W932 W942 W943 W952 W954 |
| Reduction THR dislocation | W396 W485 |
| Hip ORIF | O171 W191 W241 |
| *Non-joint specific procedure codes* |  |
| Dislocation reduction | W913 W66 W65 W919 W911 |
| Washout/debridement | W80 W81 S571 T622 T963 W336 |
| Revision | W582 W580 Y032 |
| ORIF | W19 W20 W22 W23 W24 W25 W26 W65 W66 W67 W281 |
| EUA | W924 W923 |
| Girdlestone | T572 |
|  |  |
| *Knee* |  |
| Location | Z846 Z845 Z844 Z765 Z771 Z774 Z787 O132 |
| *Joint-specific procedure codes* |  |
| Other open/arthroscopic knee procedures | O18 W40 W41 W42 W421 T593 T603 W426 W783 W843 O192 W70 W85 W87 W84 W82 O18 W40 W41 W42 |
| Above-knee amputation | X093 |
| *Non-joint specific procedure codes* |  |
| MUA | W91 W78 |
| Dislocation | W66 W67 W659 |
| Washout / debridement | W80 W81 W852 S571 T622 T963 W336 W69 |
| Revision | W582 Y032 |
| ORIF | W19 W20 W21 W22 W23 W24 W25 W26 W28W65 W67 O17 |
| EUA | W924 W923 |

Figure S6 - OPCS-4 procedure codes and definitions.

| **Group** | **Variable** | **Source** | **Modelled as** | **Base/centre** |
| --- | --- | --- | --- | --- |
|  |  |  |  |  |
| Patient | Sex | NJR | Binary | Male |
|  | Age | NJR | Restricted cubic splines | 65 |
|  | ASA grade | NJR | Categorical: I, II, III, IV-V (excluded) | I |
|  | Charlson Comorbidity Index | HES | Categorical: none (0), mild (1-2), moderate (3-4), severe (5+) | None (0) |
|  | Obesity | NJR, HES | Binary | Absent |
|  | Mental health diagnosis | HES | Binary | Absent |
|  | Smoking | HES | Binary | Absent |
|  | Hospital admissions in past year | HES | Categorial: 0, 1, 2+ | 0 |
|  | Previous contralateral surgery | NJR, HES | Binary | No |
|  |  |  |  |  |
| Socioeconomic | Ethnicity | HES | Categorical: white, non-white, unspecified | White |
|  | Residence | HES | Categorical: rural, urban, unknown | Urban |
|  | Index of Multiple Deprivation quintile | HES | Categorical: least deprived, 2, 3, 4, most deprived | Least deprived |
|  |  |  |  |  |
| Surgical | Indication | NJR | Categorical: osteoarthritis (OA) only, OA plus other, other only | OA only |
|  | Compartment (UKR) | NJR | Categorical: medial, lateral, patellofemoral, unspecified | Medial |
|  | Surgical approach (THR) | NJR | Categorical: posterior, hardinge/anterolateral/lateral, anterior, trochanteric osteotomy, other | Posterior |
|  | Surgical approach (TKR/UKR) | NJR | Categorical: medial parapatellar, mid-vastus, sub-vastus, lateral parapatellar, other | Medial parapatellar |
|  | Implant fixation | NJR | Categorical: cemented, uncemented, hybrid, reverse hybrid, unspecified | Cemented |
|  | Bearing (THR) | NJR | Categorical: MoP, CoP, CoC, MoM, CoM, dual mobility, unclassified | MoP |
|  | Bearing (TKR) | NJR | Categorical: unconstrained fixed, PS fixed, unconstrained mobile, PS mobile, constrained condylar, unclassified | Unconstrained fixed |
|  | Bearing (UKR) | NJR | Categorical: mobile, fixed, unclassified | Mobile |
|  | Femoral head (THR) | NJR | Restricted cubic splines | 32 |
|  | Anaesthetic | NJR | Categorical: GA, GA and neuraxial, neuraxial, nerve block only | Neuraxial |
|  | Nerve block given | NJR | Binary | No |
|  | VTE prophylaxis | NJR | Categorical: chemical and mechanical, chemical only, mechanical only, none | Chemical and mechanical |
|  |  |  |  |  |
| Unit | Unit sector | NJR | Binary: NHS / independent | NHS |
|  | Cumulative day case procedure volume (procedure specific) | NJR | Restricted cubic splines | Median for procedures in 2022 |
|  | Date of surgery | NJR | Restricted cubic splines | 1st February 2022 |
|  |  |  |  |  |

Figure S7 - Table of variables included in flexible parametric survival models. ASA = American Society of Anaesthesiologists; NHS = National Health Service; THR = Total Hip Replacement; TKR = Total Knee Replacement; UKR = Unicompartmental Knee Replacement; MoP = Metal-on-polyethylene; CoP = Ceramic-on-polyethylene; CoC = Ceramic-on-ceramic; MoM = Metal-on-metal; CoM = Ceramic-on-metal; GA = General Anaesthetic; VTE = Venous Thromboembolism.


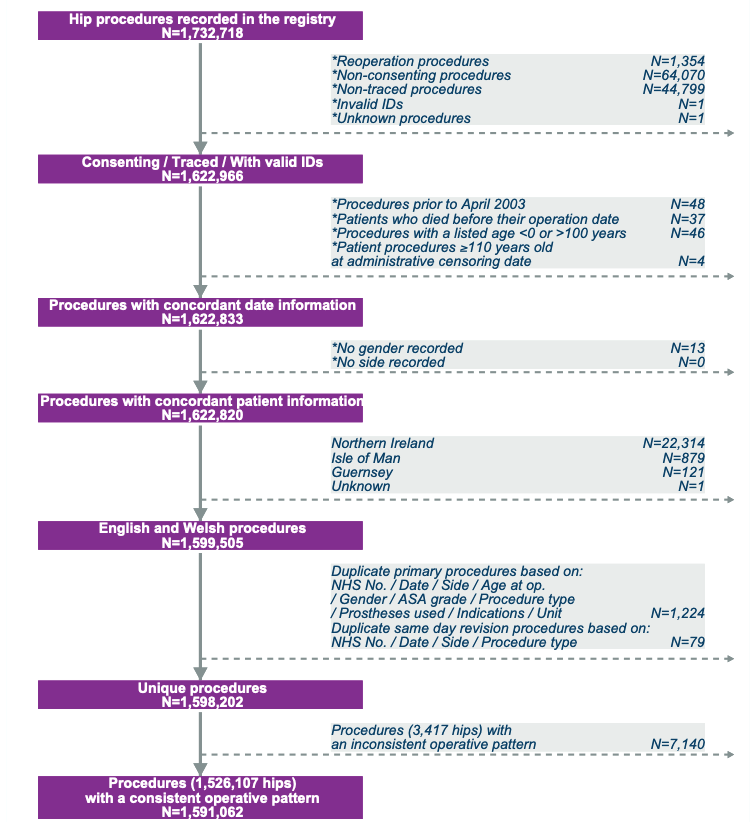


Figure S8 – National Joint Registry data cleaning process flowchart for hip procedures, taken from NJR 20^th^ Annual report, 2023.


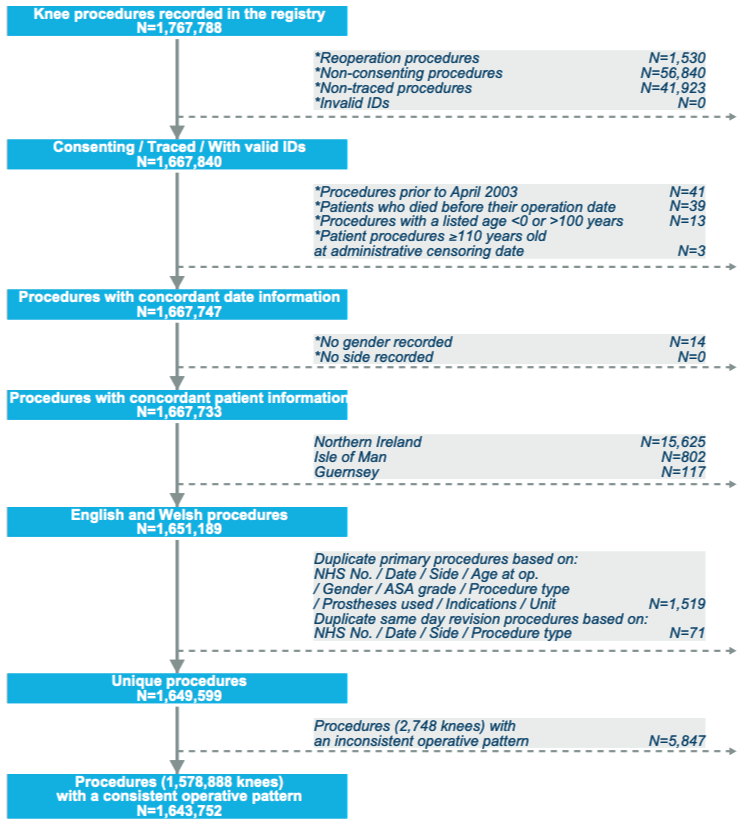


Figure S9 - National Joint Registry data cleaning process flowchart for knee procedures, taken from NJR 20^th^ Annual report, 2023.


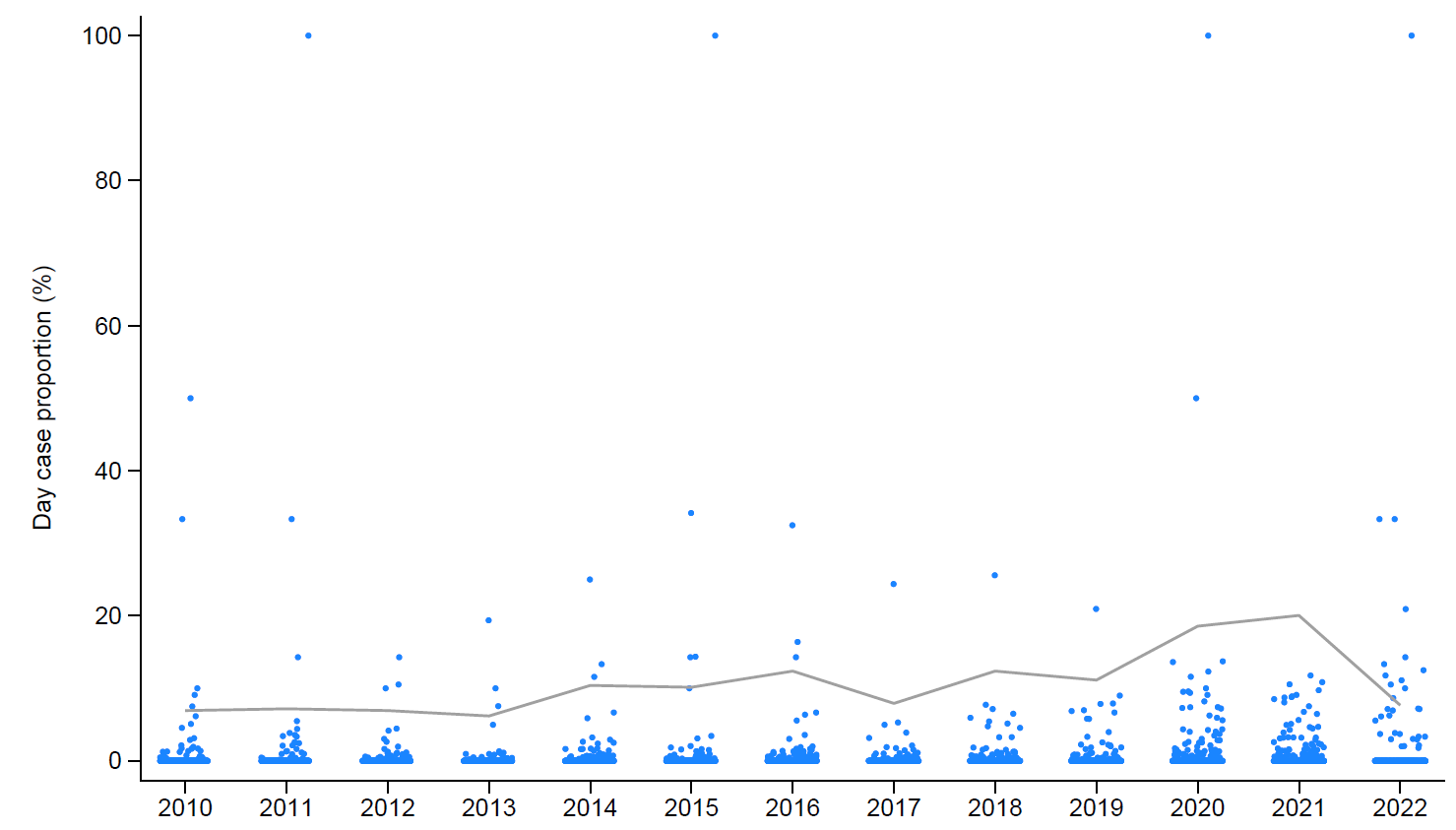


Figure S10 – Total hip replacement individual unit day case proportion, by year, for 404 active units within the study period. Each dot represents a unit. The line represents the proportion of active units performing at least one day case THR that year. Note 2022 is incomplete; data ends March 31^st^.


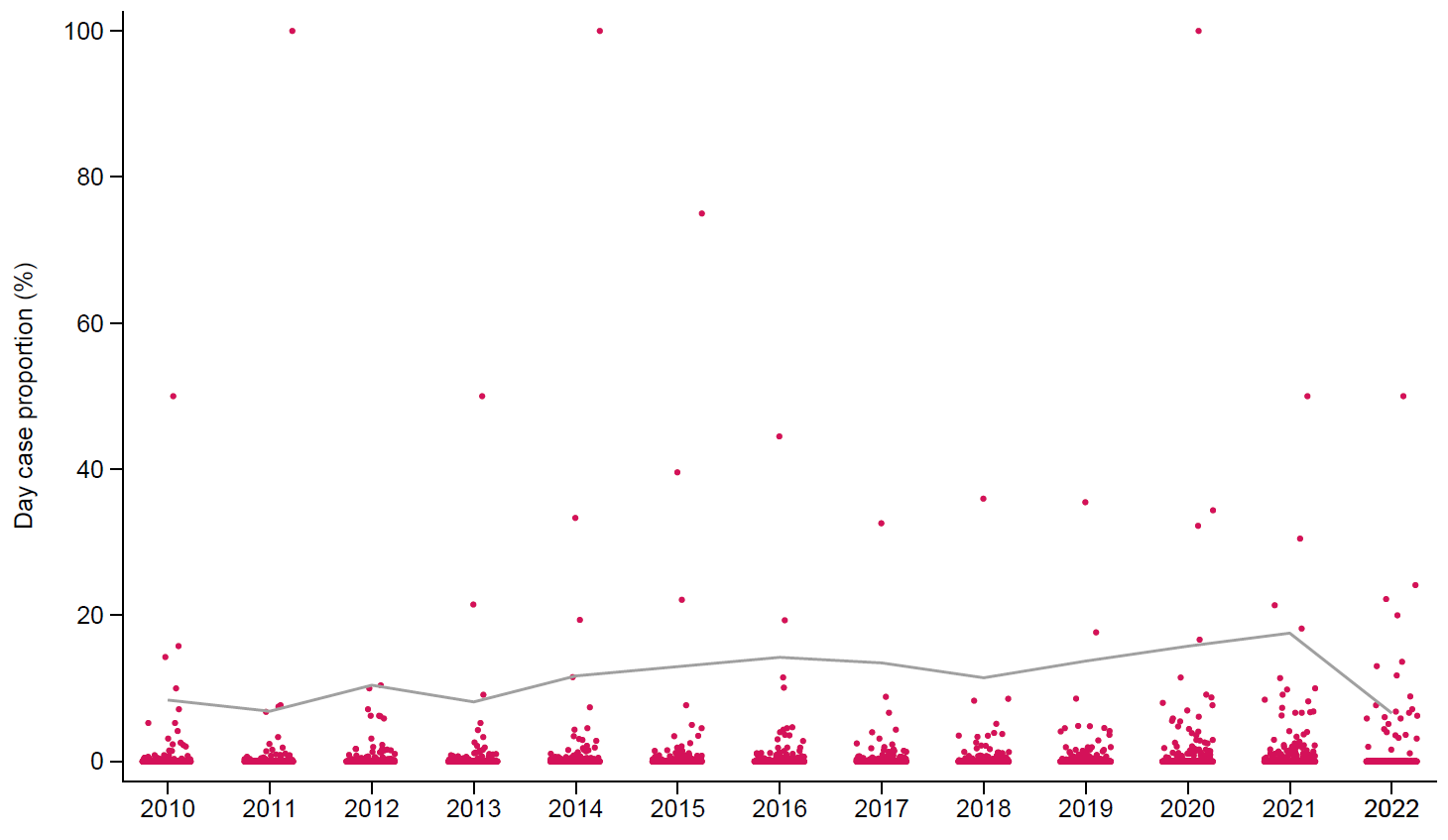


Figure S11 - Total knee replacement individual unit day case proportion, by year, for 393 active units within the study period. Each dot represents a unit. The line represents the proportion of active units performing at least one day case TKR that year. Note 2022 is incomplete; data ends March 31^st^.


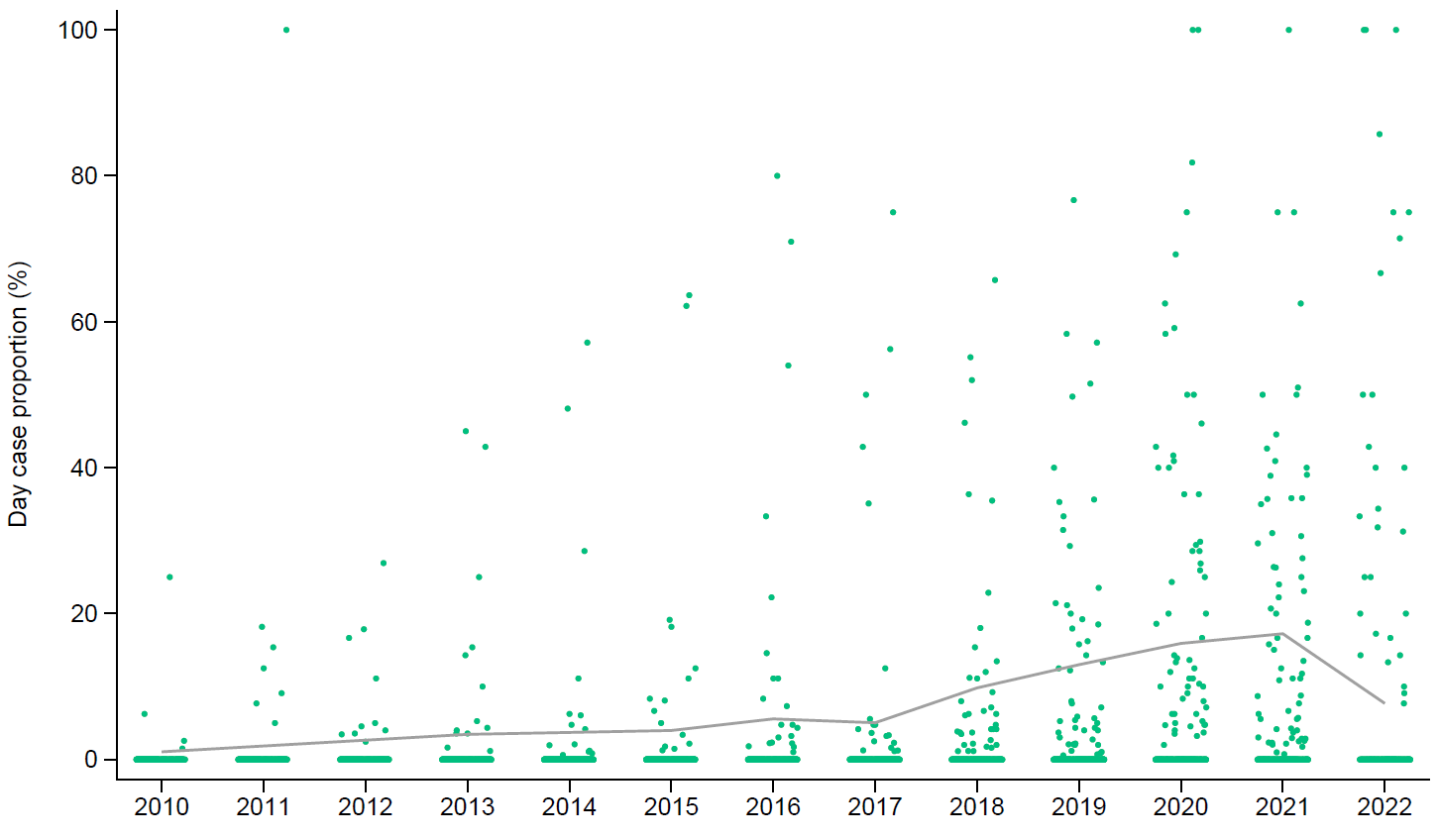


Figure S12 - Unicompartmental knee replacement individual unit day case proportion, by year, for 377 active units within the study period. Each dot represents a unit. The line represents the proportion of active units performing at least one day case UKR that year. Note 2022 is incomplete; data ends March 31^st^.


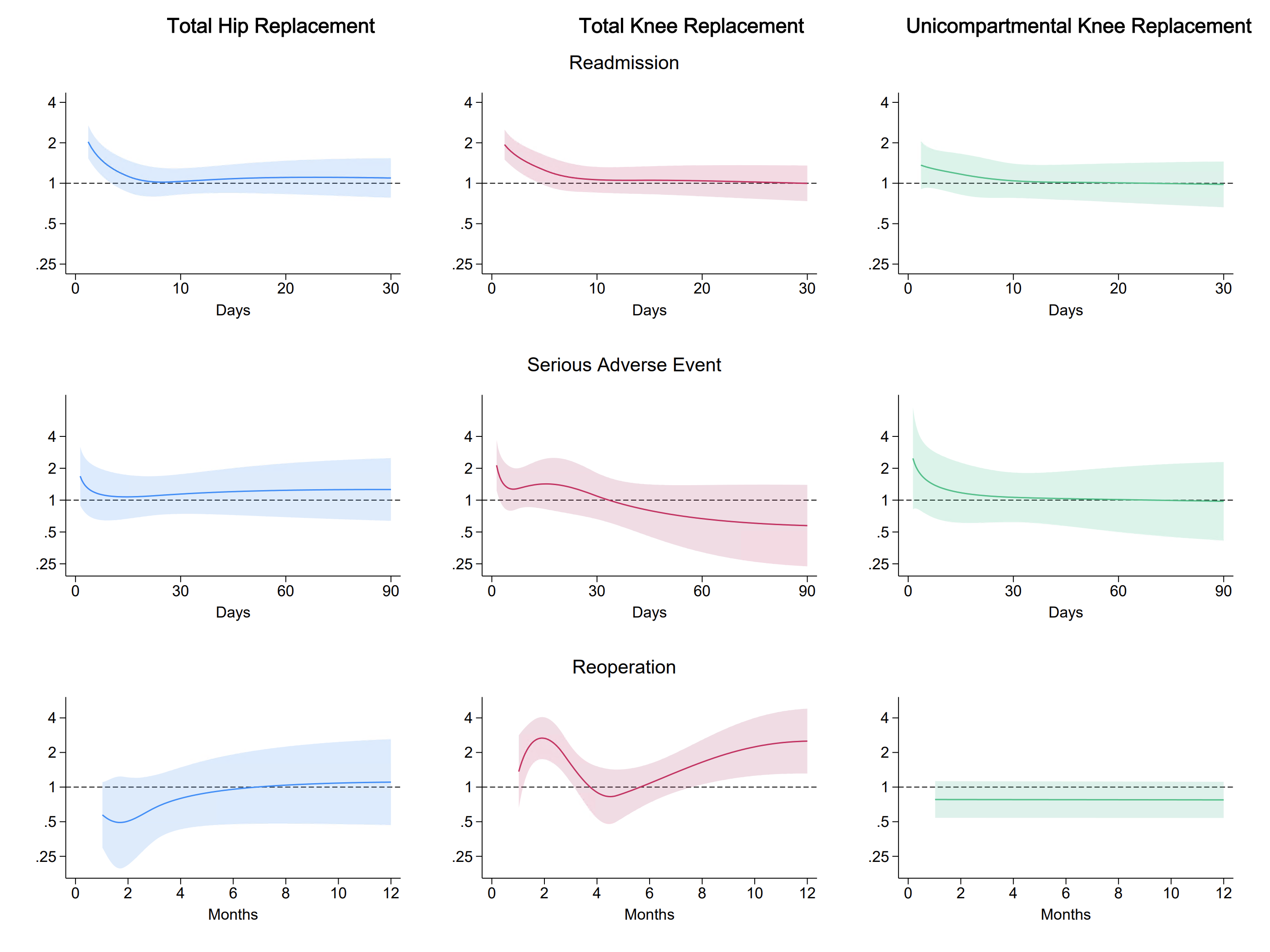


Figure S13 - Adjusted hazard ratios over time for day case compared to one-day inpatient joint replacement, generated from standardised survival probabilities using flexible parametric survival models, for each procedure. Shaded areas represent 95% confidence intervals; the effect of the exposure is not statistically significant at that time-point when these cross 1 (dashed line). The majority of the outcomes show significant time-varying effects.

|  | | |  | **Inpatient (LOS = 1)** | | |
| --- | --- | --- | --- | --- | --- | --- |
| **Diagnosis** | **n** | **%** |  | **Diagnosis** | **n** | **%** |
| *Total Hip Replacement* |  |  |  |  |  |  |
| R89 Abnormal findings in specimens from other organs, systems and tissues | 13 | 8.6 |  | M79.86 Other specified soft tissue disorders (Lower leg) | 154 | 11.3 |
| M79.86 Other specified soft tissue disorders (Lower leg) | 12 | 7.9 |  | T84.0 Mechanical complication of internal joint prosthesis | 132 | 9.7 |
| I48.9 Atrial fibrillation and atrial flutter, unspecified | 8 | 5.3 |  | T84.5 Infection and inflammatory reaction due to internal joint prosthesis | 53 | 3.9 |
| R33 Retention of urine | 7 | 4.6 |  | M25.55 Pain in joint (Pelvic region and thigh) | 41 | 3.0 |
| T84.0 Mechanical complication of internal joint prosthesis | 7 | 4.6 |  | T84.8 Other complications of internal orthopaedic prosthetic devices, implants and grafts | 39 | 2.9 |
| M79.66 Pain in limb (Lower leg) | 5 | 3.3 |  | T81.0 Haemorrhage and haematoma complicating a procedure, not elsewhere classified | 34 | 2.5 |
| T81.4 Infection following a procedure, not elsewhere classified | 4 | 2.6 |  | M79.66 Pain in limb (Lower leg) | 34 | 2.5 |
| R07.4 Chest pain, unspecified | 3 | 2.0 |  | R60.0 Localised oedema | 28 | 2.1 |
| R00.1 Bradycardia, unspecified | 3 | 2.0 |  | M79.80 Other specified soft tissue disorders (Multiple sites) | 28 | 2.1 |
| M25.55 Pain in joint (Pelvic region and thigh) | 3 | 2.0 |  | J18.1 Lobar pneumonia, unspecified | 27 | 2.0 |
|  |  |  |  |  |  |  |
| *Total Knee Replacement* |  |  |  |  |  |  |
| T84.8 Other complications of internal orthopaedic prosthetic devices, implants and grafts | 12 | 6.4 |  | M79.86 Other specified soft tissue disorders (Lower leg) | 123 | 9.7 |
| R89 Abnormal findings in specimens from other organs, systems and tissues | 12 | 6.4 |  | T84.8 Other complications of internal orthopaedic prosthetic devices, implants and grafts | 69 | 5.5 |
| M79.86 Other specified soft tissue disorders (Lower leg) | 11 | 5.9 |  | M79.66 Pain in limb (Lower leg) | 64 | 5.1 |
| I26.9 Pulmonary embolism without mention of acute cor pulmonale | 8 | 4.3 |  | M25.56 Pain in joint (Lower leg) | 59 | 4.7 |
| R33 Retention of urine | 7 | 3.7 |  | T84.5 Infection and inflammatory reaction due to internal joint prosthesis | 51 | 4.0 |
| T81.0 Haemorrhage and haematoma complicating a procedure, not elsewhere classified | 5 | 2.7 |  | L03.1 Cellulitis of other parts of limb | 42 | 3.3 |
| M79.66 Pain in limb (Lower leg) | 5 | 2.7 |  | I26.9 Pulmonary embolism without mention of acute cor pulmonale | 41 | 3.2 |
| I80.2 Phlebitis and thrombophlebitis of other deep vessels of lower extremities | 5 | 2.7 |  | I80.2 Phlebitis and thrombophlebitis of other deep vessels of lower extremities | 37 | 2.9 |
| J18.1 Lobar pneumonia, unspecified | 4 | 2.1 |  | T81.4 Infection following a procedure, not elsewhere classified | 33 | 2.6 |
| M25.46 Effusion of joint (Lower leg) | 4 | 2.1 |  | T81.0 Haemorrhage and haematoma complicating a procedure, not elsewhere classified | 30 | 2.4 |
|  |  |  |  |  |  |  |
| *Unicompartmental knee replacement* |  |  |  |  |  |  |
| T81.0 Haemorrhage and haematoma complicating a procedure, not elsewhere classified | 12 | 9.7 |  | T84.8 Other complications of internal orthopaedic prosthetic devices, implants and grafts | 28 | 7.1 |
| T84.8 Other complications of internal orthopaedic prosthetic devices, implants and grafts | 11 | 8.9 |  | M79.86 Other specified soft tissue disorders (Lower leg) | 24 | 6.1 |
| M25.56 Pain in joint (Lower leg) | 7 | 5.6 |  | M79.66 Pain in limb (Lower leg) | 19 | 4.8 |
| T81.4 Infection following a procedure, not elsewhere classified | 6 | 4.8 |  | M25.56 Pain in joint (Lower leg) | 19 | 4.8 |
| M25.46 Effusion of joint (Lower leg) | 5 | 4.0 |  | M25.46 Effusion of joint (Lower leg) | 16 | 4.1 |
| M79.86 Other specified soft tissue disorders (Lower leg) | 5 | 4.0 |  | T84.5 Infection and inflammatory reaction due to internal joint prosthesis | 15 | 3.8 |
| R07.4 Chest pain, unspecified | 4 | 3.2 |  | T81.0 Haemorrhage and haematoma complicating a procedure, not elsewhere classified | 12 | 3.1 |
| M79.66 Pain in limb (Lower leg) | 4 | 3.2 |  | L03.1 Cellulitis of other parts of limb | 11 | 2.8 |
| I26.9 Pulmonary embolism without mention of acute cor pulmonale | 4 | 3.2 |  | T81.4 Infection following a procedure, not elsewhere classified | 9 | 2.3 |
| R11 Nausea and vomiting | 3 | 2.4 |  | I26.9 Pulmonary embolism without mention of acute cor pulmonale | 8 | 2.0 |

Figure S14 - Most common primary ICD-10 diagnosis codes and definitions for patients readmitted within 30 days, by THR, TKR, UKR. The left column represents day case patients, the right column represents inpatients. Percentage is ICD-10 code proportion within the 30-day readmissions.

Figure S15- Adjusted probabilities for mortality following day case compared to one-day inpatient joint replacement, generated using standardised survival estimates from flexible parametric survival models. Coloured lines represent day cases, and grey dashed lines represent the inpatient comparison group. Blue = THR, red = TKR, green = UKR. Shaded areas represent 95% confidence intervals. These overlap for all procedures.


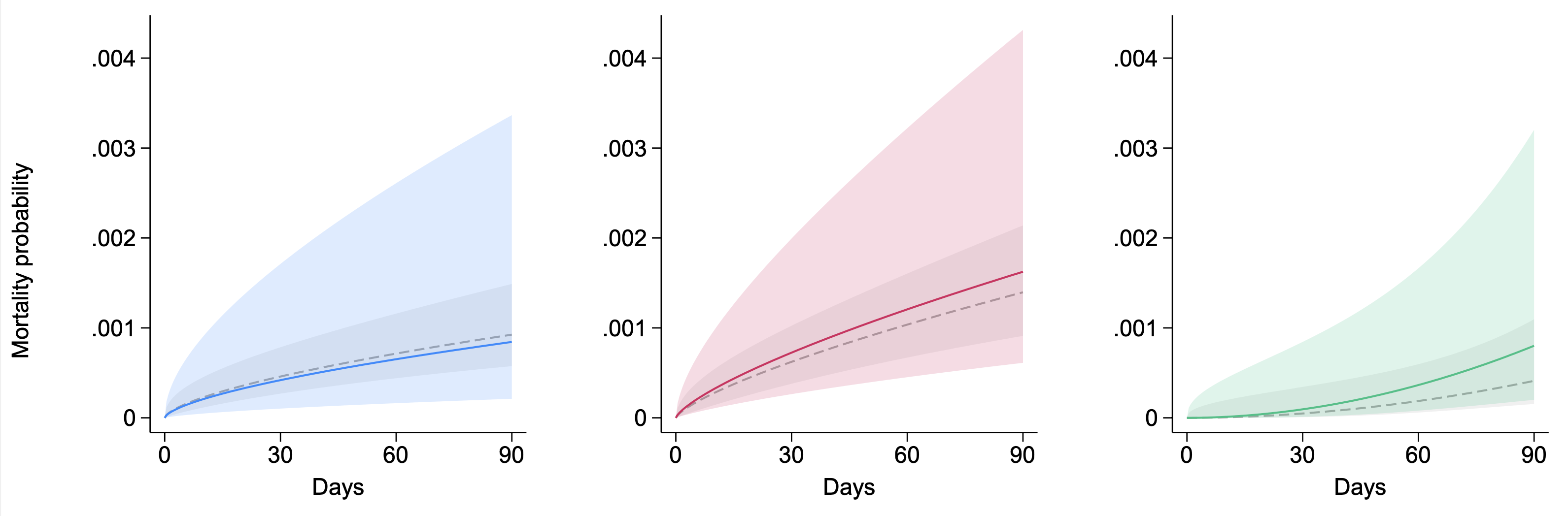

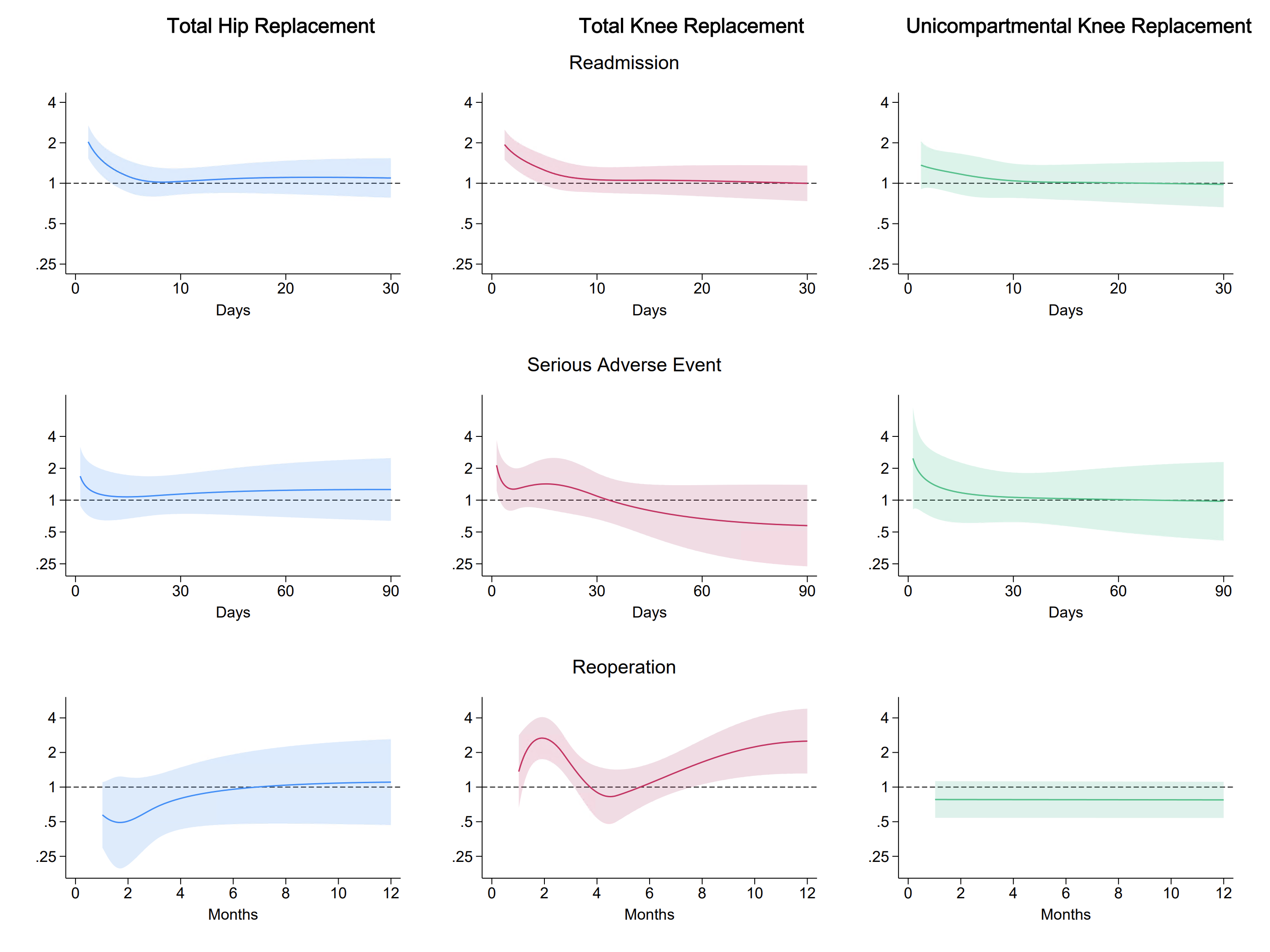


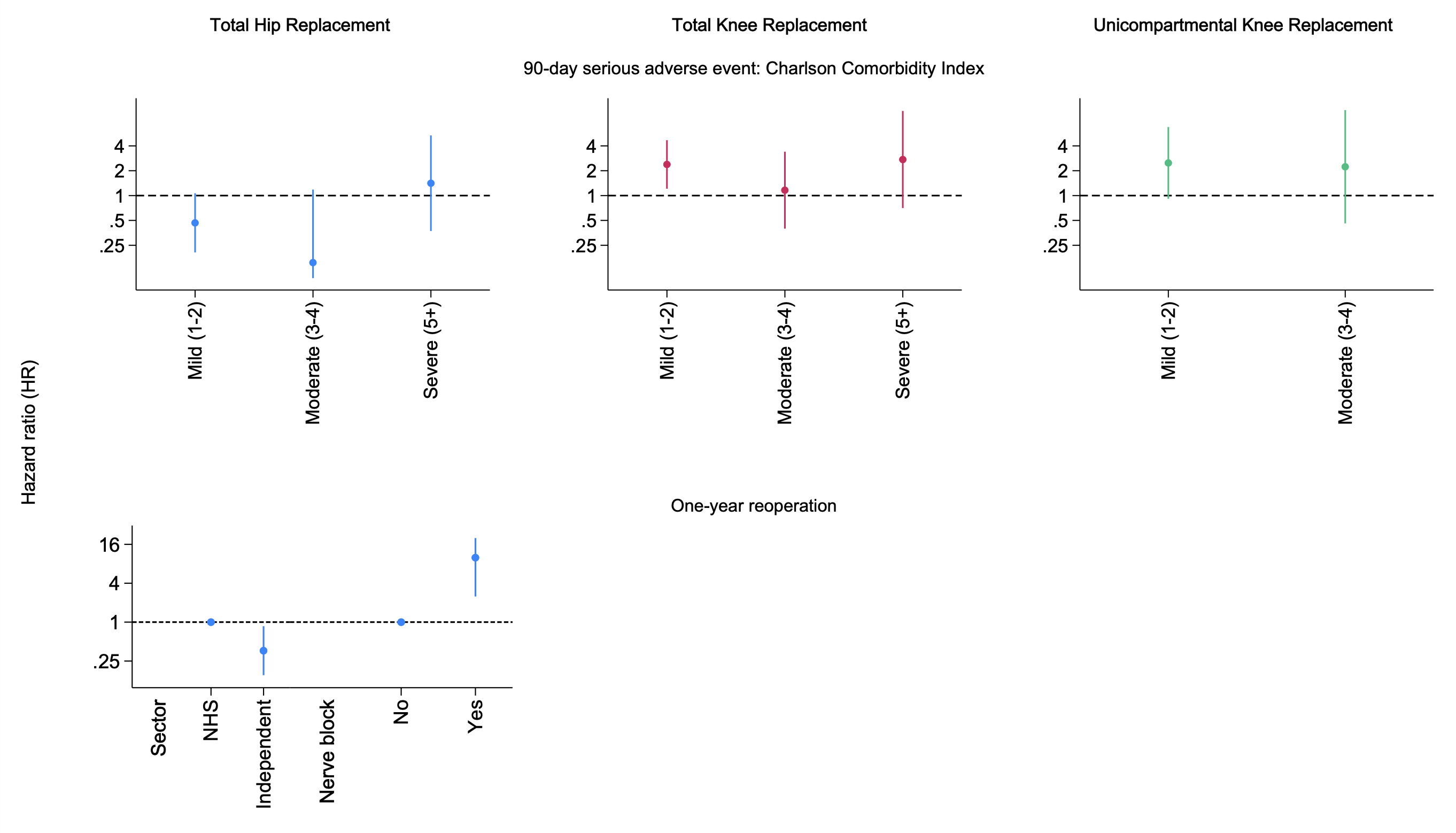


Figure S16 – Day case surgery-associated risk factors for 90-day serious adverse events (top row) and one-year reoperations (bottom row) for each procedure. Blue = THR, red = TKR, green = UKR. Bars represent 95% confidence intervals.

|  | **Day Case** | **Inpatient (LOS=1)** | **p=** |
| --- | --- | --- | --- |
|  |  |  |  |
| *Total Hip Replacement* | |  |  |
| Pain | 0 (0.0%) | 6 (0.0%) | 0.481 |
| Infection | 8 (0.3%) | 107 (0.4%) | 0.786 |
| Other | 0 (0.0%) | 9 (0.0%) | 0.389 |
| Aseptic loosening | 2 (0.1%) | 15 (0.1%) | 0.520 |
| Lysis | 0 (0.0%) | 2 (0.0%) | 0.684 |
| PPF | 2 (0.1%) | 30 (0.1%) | 0.769 |
| Implant fracture | 0 (0.0%) | 6 (0.0%) | 0.481 |
| Implant wear | 1 (0.0%) | 4 (0.0%) | 0.297 |
| Head/socket mismatch | 0 (0.0%) | 1 (0.0%) | 0.774 |
| Malalignment | 2 (0.1%) | 10 (0.0%) | 0.238 |
| ARMD | 0 (0.0%) | 0 (0.0%) | - |
| Total | 17 (0.7%) | 218 (0.7%) | 0.820 |
|  |  |  |  |
| *Total Knee Replacement* | |  |  |
| Infection | 9 (0.4%) | 50 (0.2%) | 0.228 |
| Dislocation/subluxation | 1 (0.0%) | 0 (0.0%) | 0.003 |
| Pain | 0 (0.0%) | 4 (0.0%) | 0.494 |
| Malalignment | 0 (0.0%) | 1 (0.0%) | 0.733 |
| PPF | 0 (0.0%) | 3 (0.0%) | 0.554 |
| Stiffness | 0 (0.0%) | 5 (0.0%) | 0.445 |
| Progressive arthritis remaining knee | 0 (0.0%) | 1 (0.0%) | 0.733 |
| Instability | 1 (0.0%) | 5 (0.0%) | 0.619 |
| Implant wear | 0 (0.0%) | 0 (0.0%) | - |
| Other | 4 (0.2%) | 2 (0.0%) | <0.001 |
| Aseptic loosening/lysis | 0 (0.0%) | 4 (0.0%) | 0.494 |
| Total | 14 (0.6%) | 70 (0.3%) | 0.063 |
|  |  |  |  |
| *Unicompartmental Knee Replacement* | | |  |
| Infection | 3 (0.1%) | 12 (0.1%) | 0.977 |
| Dislocation/subluxation | 3 (0.1%) | 14 (0.1%) | 0.786 |
| Pain | 0 (0.0%) | 4 (0.0%) | 0.313 |
| Malalignment | 1 (0.0%) | 3 (0.0%) | 0.815 |
| PPF | 1 (0.0%) | 8 (0.1%) | 0.493 |
| Stiffness | 0 (0.0%) | 0 (0.0%) | - |
| Progressive arthritis remaining knee | 0 (0.0%) | 5 (0.0%) | 0.259 |
| Instability | 1 (0.0%) | 3 (0.0%) | 0.815 |
| Implant wear | 0 (0.0%) | 11 (0.1%) | 0.094 |
| Other | 5 (0.2%) | 6 (0.1%) | 0.038 |
| Aseptic loosening/lysis | 1 (0.0%) | 15 (0.1%) | 0.162 |
| Total | 14 (0.5%) | 74 (0.7%) | 0.305 |

Figure S17 - Indications for revisions recorded in NJR, by joint. Note indications not mutually exclusive. Percentages given as a proportion of all cases per joint. P-values generated by chi-square tests. LOS = Length of Stay; PPF = Peri-prosthetic Fracture; ARMD = Adverse Reaction to Metal Debris.

| **OPCS-4 code** | **Procedure** | **n** | **%** |
| --- | --- | --- | --- |
|  |  |  |  |
| *Total Hip Replacement* | |  |  |
| W396 | Closed reduction of dislocated total prosthetic replacement of hip joint | 128 | 48.9 |
| W913 | Manipulation of prosthetic joint NEC | 36 | 13.7 |
| W801 | Open debridement and irrigation of joint | 10 | 3.8 |
| W201 | Primary open reduction of fracture of long bone and extramedullary fixation using plate NEC | 8 | 3.1 |
| W803 | Open irrigation of joint NEC | 7 | 2.7 |
| W669 | Unspecified primary closed reduction of traumatic dislocation of joint | 7 | 2.7 |
| T622 | Excision of bursa NEC | 4 | 1.5 |
| W394 | Attention to total prosthetic replacement of hip joint NEC | 4 | 1.5 |
| W246 | Closed reduction of fracture of bone and fixation using nail or screw | 4 | 1.5 |
| W383 | Revision of total prosthetic replacement of hip joint not using cement | 3 | 1.1 |
|  |  |  |  |
| *Total Knee Replacement* | |  |  |
| W913 | Manipulation of prosthetic joint NEC | 341 | 60.2 |
| W919 | Unspecified other manipulation of joint | 26 | 4.6 |
| W871 | Diagnostic endoscopic examination of knee joint and biopsy of lesion of knee joint | 12 | 2.1 |
| W426 | Arthrolysis of total prosthetic replacement of knee joint | 12 | 2.1 |
| W201 | Primary open reduction of fracture of long bone and extramedullary fixation using plate NEC | 12 | 2.1 |
| W693 | Partial synovectomy | 11 | 1.9 |
| W801 | Open debridement and irrigation of joint | 10 | 1.8 |
| W803 | Open irrigation of joint NEC | 8 | 1.4 |
| S571 | Debridement of skin NEC | 8 | 1.4 |
| W802 | Open debridement of joint NEC | 7 | 1.2 |
|  |  |  |  |
| *Unicompartmental Knee Replacement* | |  |  |
| W822 | Endoscopic resection of semilunar cartilage NEC | 75 | 21.0 |
| W851 | Endoscopic removal of loose body from knee joint | 36 | 10.0 |
| W913 | Manipulation of prosthetic joint NEC | 33 | 9.0 |
| W802 | Open debridement of joint NEC | 12 | 3.0 |
| W879 | Unspecified diagnostic endoscopic examination of knee joint | 12 | 3.0 |
| W693 | Partial synovectomy | 11 | 3.0 |
| W783 | Release of contracture of knee joint | 10 | 3.0 |
| W891 | Endoscopic chondroplasty NEC | 10 | 3.0 |
| W835 | Endoscopic articular thermal chondroplasty | 9 | 3.0 |
| W803 | Open irrigation of joint NEC | 8 | 2.0 |

Figure S18 - Most common primary OPCS-4 codes for non-revision reoperations identified through HES by THR, TKR, UKR. Percentage is OPCD-4 code proportion of patients who had a reoperation within one year. NEC = Not elsewhere classifiable.

| **Model** | **Adjusting for** | **Inpatient (LOS 1) absolute risk (%)** | | | **Day case (LOS 0) absolute risk (%)** | | | | | **Absolute risk difference (%)** | | | | **Relative risk** | | | |
| --- | --- | --- | --- | --- | --- | --- | --- | --- | --- | --- | --- | --- | --- | --- | --- | --- | --- |
|  |  |  |  |  | | |  | |  | | | |  | | | |  |
| *30-day readmission* | |  |  |  | | |  | |  | | | |  | | | |  |
| Model 1 | Base | 4.67 [4.43, 4.92] | | 6.16 [5.27, 7.20] | | | | 1.49 [0.50, 2.49] | | | 1.32 [1.12, 1.56] | | | |  |  |  |
| Model 2 | Patient characteristics | 4.49 [4.25, 4.74] | | 6.18 [5.29, 7.22] | | | | 1.69 [0.70, 2.68] | | | 1.38 [1.17, 1.62] | | | |  |  |  |
| Model 3 | Patient, socioeconomic characteristics | 4.50 [4.26, 4.75] | | 6.18 [5.29, 7.22] | | | | 1.69 [0.70, 2.68] | | | 1.38 [1.17, 1.62] | | | |  |  |  |
| Model 4 | Patient, socioeconomic, surgical characteristics | 4.61 [4.34, 4.89] | | 6.17 [5.29, 7.21] | | | | 1.57 [0.57, 2.56] | | | 1.34 [1.14, 1.58] | | | |  |  |  |
| Model 5 | Patient, socioeconomic, surgical, unit characteristics | 4.65 [4.26, 5.07] | | 6.18 [5.29, 7.21] | | | | 1.53 [0.49, 2.57] | | | 1.33 [1.11, 1.59] | | | |  |  |  |
| **Model 6** | **Final model - Patient, socioeconomic, surgical, unit characteristics with interactions** | **4.85 [4.40, 5.33]** | | **6.18 [5.31, 7.19]** | | | | **1.33 [0.29, 2.38]** | | | **1.28 [1.07, 1.53]** | | | |  |  |  |
| Sensitivity 1 | Base, including cases with missing data | 4.65 [4.41, 4.90] | | 6.24 [5.35, 7.29] | | | | 1.60 [0.60, 2.59] | | | 1.34 [1.14, 1.58] | | | |  |  |  |
| Sensitivity 2 | Unit and surgeon clustering (multilevel logistic regression model) | - |  | - | | |  | | - | | | | 1.40* [1.16, 1.69] | | | |  |
|  |  |  |  |  | | |  | |  | | | |  | | | |  |
| *90-day serious adverse event* | |  | |  | | |  | |  | | | |  | | | |  |
| Model 1 | Base | 1.17 [1.05, 1.31] | | 1.40 [1.01, 1.96] | |  | | 0.22 [-0.27, 0.71] | | | | 1.19 [0.84, 1.70] | | | |  |  |
| Model 2 | Patient characteristics | 1.09 [0.97, 1.22] | | 1.41 [1.01, 1.97] | | | | 0.32 [-0.17, 0.81] | | | 1.29 [0.91, 1.84] | | | |  |  |  |
| Model 3 | Patient, socioeconomic characteristics | 1.09 [0.97, 1.22] | | 1.41 [1.01, 1.97] | | | | 0.32 [-0.17, 0.81] | | | 1.29 [0.91, 1.84] | | | |  |  |  |
| Model 4 | Patient, socioeconomic, surgical characteristics | 1.15 [1.01, 1.30] | | 1.41 [1.01, 1.97] | | | | 0.26 [-0.23, 0.76] | | | 1.23 [0.86, 1.76] | | | |  |  |  |
| Model 5 | Patient, socioeconomic, surgical, unit characteristics | 1.14 [0.94, 1.38] | | 1.41 [1.01, 1.97] | | | | 0.27 [-0.25, 0.80] | | | 1.24 [0.84, 1.83] | | | |  |  |  |
| **Model 6** | **Final model - Patient, socioeconomic, surgical, unit characteristics with interactions** | **1.13 [0.93, 1.37]** | | **1.42 [1.02, 1.99]** | | | | **0.29 [-0.23, 0.82]** | | | **1.26 [0.85, 1.85]** | | | |  |  |  |
| Sensitivity 1 | Base, including cases with missing data | 1.17 [1.05, 1.30] | | 1.42 [1.02, 1.98] | | | | 0.25 [-0.24, 0.74] | | | 1.22 [0.86, 1.73] | | | |  |  |  |
| Sensitivity 2 | Unit and surgeon clustering (multilevel logistic regression model) | - |  | - | | |  | | - | | | | 1.22* [0.84, 1.76] | | | |  |
|  |  |  |  |  | | |  | |  | | | |  | | | |  |
| *One-year reoperation* | |  |  |  | | |  | |  | | | |  | | | |  |
| Model 1 | Base | 1.49 [1.36, 1.65] | | 1.26 [0.88, 1.82] | | | | -0.23 [-0.71, 0.25] | | | 0.85 [0.58, 1.23] | | | |  |  |  |
| Model 2 | Patient characteristics | 1.37 [1.24, 1.52] | | 1.27 [0.88, 1.82] | | | | -0.11 [-0.58, 0.37] | | | 0.92 [0.63, 1.34] | | | |  |  |  |
| Model 3 | Patient, socioeconomic characteristics | 1.37 [1.24, 1.52] | | 1.27 [0.88, 1.82] | | | | -0.11 [-0.59, 0.37] | | | 0.92 [0.63, 1.34] | | | |  |  |  |
| Model 4 | Patient, socioeconomic, surgical characteristics | 1.39 [1.25, 1.55] | | 1.26 [0.88, 1.81] | | | | -0.12 [-0.61, 0.36] | | | 0.91 [0.62, 1.33] | | | |  |  |  |
| Model 5 | Patient, socioeconomic, surgical, unit characteristics | 1.43 [1.21, 1.68] | | 1.26 [0.88, 1.81] | | | | -0.17 [-0.68, 0.34] | | | 0.88 [0.59, 1.31] | | | |  |  |  |
| **Model 6** | **Final model - Patient, socioeconomic, surgical, unit characteristics with interactions** | **1.51 [1.27, 1.81]** | | **1.26 [0.89, 1.79]** | | | | **-0.25 [-0.77, 0.27]** | | | **0.83 [0.56, 1.24]** | | | |  |  |  |
| Sensitivity 1 | Base, including cases with missing data | 1.48 [1.34, 1.63] | | 1.29 [0.90, 1.84] | | | | -0.19 [-0.67, 0.29] | | | 0.87 [0.60, 1.26] | | | |  |  |  |
| Sensitivity 2 | Unit and surgeon clustering (multilevel logistic regression model) | - |  | - | | |  | | - | | | | 0.90* [0.61, 1.33] | | | |  |

Figure S19 - Summary of flexible parametric survival model building for THR. Estimates were generated using standardised survival functions, with covariates standardised to those of day case patients. See Table 5 for details of the individual variables comprising each covariate group. *=Odds ratio rather than RR.

| **Model** | **Adjusting for** | **Inpatient (LOS 1) absolute risk (%)** | | | **Day case (LOS 0) absolute risk (%)** | | | | | **Absolute risk difference (%)** | | | | **Relative risk** | | | |
| --- | --- | --- | --- | --- | --- | --- | --- | --- | --- | --- | --- | --- | --- | --- | --- | --- | --- |
|  |  |  |  |  | | |  | |  | | | |  | | | |  |
| *30-day readmission* | |  |  |  | | |  | |  | | | |  | | | |  |
| Model 1 | Base | 5.90 [5.60, 6.23] | | 7.58 [6.61, 8.69] | | | | 1.68 [0.59, 2.76] | | | 1.28 [1.11, 1.49] | | | |  |  |  |
| Model 2 | Patient characteristics | 5.65 [5.35, 5.98] | | 7.60 [6.64, 8.71] | | | | 1.95 [0.87, 3.03] | | | 1.34 [1.16, 1.56] | | | |  |  |  |
| Model 3 | Patient, socioeconomic characteristics | 5.69 [5.38, 6.02] | | 7.60 [6.64, 8.71] | | | | 1.91 [0.83, 2.99] | | | 1.34 [1.15, 1.55] | | | |  |  |  |
| Model 4 | Patient, socioeconomic, surgical characteristics | 5.63 [5.30, 5.98] | | 7.59 [6.63, 8.69] | | | | 1.96 [0.88, 3.04] | | | 1.35 [1.16, 1.56] | | | |  |  |  |
| Model 5 | Patient, socioeconomic, surgical, unit characteristics | 5.58 [5.08, 6.14] | | 7.60 [6.64, 8.70] | | | | 2.02 [0.86, 3.18] | | | 1.36 [1.15, 1.61] | | | |  |  |  |
| **Model 6** | **Final model - Patient, socioeconomic, surgical, unit characteristics with interactions** | **5.91 [5.29, 6.61]** | | **7.58 [6.65, 8.65]** | | | | **1.67 [0.48, 2.87]** | | | **1.28 [1.08, 1.53]** | | | |  |  |  |
| Sensitivity 1 | Base, including cases with missing data | 5.89 [5.59, 6.22] | | 7.54 [6.58, 8.65] | | | | 1.65 [0.57, 2.73] | | | 1.28 [1.10, 1.48] | | | |  |  |  |
| Sensitivity 2 | Unit and surgeon clustering (multilevel logistic regression model) | - |  | - | | |  | | - | | | | 1.38* [1.16, 1.65] | | | |  |
|  |  |  |  |  | | |  | |  | | | |  | | | |  |
| *90-day serious adverse event* | |  | |  | | |  | |  | | | |  | | | |  |
| Model 1 | Base | 1.66 [1.50, 1.85] | | 2.10 [1.61, 2.75] | |  | | 0.44 [-0.15, 1.03] | | | | 1.26 [0.95, 1.69] | | | |  |  |
| Model 2 | Patient characteristics | 1.59 [1.43, 1.77] | | 2.13 [1.63, 2.78] | | | | 0.53 [-0.06, 1.13] | | | 1.34 [1.00, 1.78] | | | |  |  |  |
| Model 3 | Patient, socioeconomic characteristics | 1.59 [1.43, 1.77] | | 2.13 [1.63, 2.78] | | | | 0.54 [-0.06, 1.13] | | | 1.34 [1.00, 1.78] | | | |  |  |  |
| Model 4 | Patient, socioeconomic, surgical characteristics | 1.59 [1.42, 1.79] | | 2.13 [1.63, 2.78] | | | | 0.54 [-0.06, 1.14] | | | 1.34 [1.00, 1.79] | | | |  |  |  |
| Model 5 | Patient, socioeconomic, surgical, unit characteristics | 1.70 [1.40, 2.06] | | 2.13 [1.63, 2.78] | | | | 0.43 [-0.23, 1.08] | | | 1.25 [0.90, 1.74] | | | |  |  |  |
| **Model 6** | **Final model - Patient, socioeconomic, surgical, unit characteristics with interactions** | **1.72 [1.41, 2.08]** | | **2.13 [1.63, 2.77]** | | | | **0.41 [-0.25, 1.07]** | | | **1.24 [0.89, 1.72]** | | | |  |  |  |
| Sensitivity 1 | Base, including cases with missing data | 1.65 [1.49, 1.84] | | 2.09 [1.60, 2.74] | | | | 0.44 [-0.15, 1.03] | | | 1.26 [0.95, 1.69] | | | |  |  |  |
| Sensitivity 2 | Unit and surgeon clustering (multilevel logistic regression model) | - |  | - | | |  | | - | | | | 1.27* [0.92, 1.74] | | | |  |
|  |  |  |  |  | | |  | |  | | | |  | | | |  |
| *One-year reoperation* | |  |  |  | | |  | |  | | | |  | | | |  |
| Model 1 | Base | 2.42 [2.21, 2.65] | | 3.53 [2.86, 4.37] | | | | 1.11 [0.33, 1.89] | | | 1.46 [1.16, 1.84] | | | |  |  |  |
| Model 2 | Patient characteristics | 2.48 [2.26, 2.72] | | 3.52 [2.85, 4.35] | | | | 1.05 [0.27, 1.82] | | | 1.42 [1.13, 1.79] | | | |  |  |  |
| Model 3 | Patient, socioeconomic characteristics | 2.48 [2.26, 2.72] | | 3.52 [2.85, 4.35] | | | | 1.04 [0.26, 1.82] | | | 1.42 [1.13, 1.79] | | | |  |  |  |
| Model 4 | Patient, socioeconomic, surgical characteristics | 2.40 [2.18, 2.65] | | 3.53 [2.86, 4.36] | | | | 1.13 [0.35, 1.91] | | | 1.47 [1.16, 1.86] | | | |  |  |  |
| Model 5 | Patient, socioeconomic, surgical, unit characteristics | 2.39 [2.03, 2.81] | | 3.58 [2.90, 4.42] | | | | 1.19 [0.34, 2.04] | | | 1.50 [1.15, 1.96] | | | |  |  |  |
| **Model 6** | **Final model - Patient, socioeconomic, surgical, unit characteristics with interactions** | **2.39 [2.03, 2.81]** | | **3.58 [2.90, 4.42]** | | | | **1.19 [0.34, 2.04]** | | | **1.50 [1.15, 1.96]** | | | |  |  |  |
| Sensitivity 1 | Base, including cases with missing data | 2.43 [2.22, 2.66] | | 3.51 [2.84, 4.34] | | | | 1.09 [0.31, 1.86] | | | 1.45 [1.15, 1.82] | | | |  |  |  |
| Sensitivity 2 | Unit and surgeon clustering (multilevel logistic regression model) | - |  | - | | |  | | - | | | | 1.40* [1.06, 1.84] | | | |  |

Figure S20 - Summary of flexible parametric survival model building for TKR. Estimates were generated using standardised survival functions, with covariates standardised to those of day case patients. See Table 5 for details of the individual variables comprising each covariate group. *=Odds ratio rather than RR.

| **Model** | **Adjusting for** | **Inpatient (LOS 1) absolute risk (%)** | | | **Day case (LOS 0) absolute risk (%)** | | | | | **Absolute risk difference (%)** | | | | **Relative risk** | | | |
| --- | --- | --- | --- | --- | --- | --- | --- | --- | --- | --- | --- | --- | --- | --- | --- | --- | --- |
|  |  |  |  |  | | |  | |  | | | |  | | | |  |
| *30-day readmission* | |  |  |  | | |  | |  | | | |  | | | |  |
| Model 1 | Base | 3.92 [3.55, 4.31] | | 4.87 [4.10, 5.78] | | | | 0.96 [0.04, 1.87] | | | 1.24 [1.02, 1.52] | | | |  |  |  |
| Model 2 | Patient characteristics | 3.87 [3.51, 4.28] | | 4.86 [4.10, 5.77] | | | | 0.99 [0.07, 1.90] | | | 1.25 [1.03, 1.53] | | | |  |  |  |
| Model 3 | Patient, socioeconomic characteristics | 3.87 [3.51, 4.28] | | 4.86 [4.10, 5.77] | | | | 0.99 [0.07, 1.90] | | | 1.26 [1.03, 1.53] | | | |  |  |  |
| Model 4 | Patient, socioeconomic, surgical characteristics | 4.09 [3.67, 4.56] | | 4.86 [4.10, 5.76] | | | | 0.77 [-0.17, 1.71] | | | 1.19 [0.97, 1.46] | | | |  |  |  |
| Model 5 | Patient, socioeconomic, surgical, unit characteristics | 4.35 [3.78, 5.01] | | 4.86 [4.10, 5.76] | | | | 0.51 [-0.51, 1.54] | | | 1.12 [0.90, 1.39] | | | |  |  |  |
| **Model 6** | **Final model - Patient, socioeconomic, surgical, unit characteristics with interactions** | **4.36 [3.71, 5.12]** | | **4.87 [4.11, 5.77]** | | | | **0.51 [-0.57, 1.60]** | | | **1.12 [0.88, 1.41]** | | | |  |  |  |
| Sensitivity 1 | Base, including cases with missing data | 3.91 [3.54, 4.30] | | 4.85 [4.09, 5.76] | | | | 0.95 [0.03, 1.86] | | | 1.24 [1.02, 1.51] | | | |  |  |  |
| Sensitivity 2 | Unit and surgeon clustering (multilevel logistic regression model) | - |  | - | | |  | | - | | | | 1.12* [0.90, 1.41] | | | |  |
|  |  |  |  |  | | |  | |  | | | |  | | | |  |
| *90-day serious adverse event* | |  | |  | | |  | |  | | | |  | | | |  |
| Model 1 | Base | 0.89 [0.72, 1.09] | | 1.03 [0.70, 1.51] | |  | | 0.15 [-0.29, 0.58] | | | | 1.16 [0.75, 1.80] | | | |  |  |
| Model 2 | Patient characteristics | 0.90 [0.73, 1.11] | | 1.03 [0.70, 1.51] | | | | 0.13 [-0.31, 0.57] | | | 1.15 [0.74, 1.77] | | | |  |  |  |
| Model 3 | Patient, socioeconomic characteristics | 0.92 [0.74, 1.13] | | 1.03 [0.70, 1.51] | | | | 0.11 [-0.32, 0.55] | | | 1.12 [0.73, 1.74] | | | |  |  |  |
| Model 4 | Patient, socioeconomic, surgical characteristics | 0.91 [0.72, 1.15] | | 1.03 [0.70, 1.51] | | | | 0.12 [-0.33, 0.56] | | | 1.13 [0.72, 1.77] | | | |  |  |  |
| Model 5 | Patient, socioeconomic, surgical, unit characteristics | 0.85 [0.62, 1.16] | | 1.03 [0.70, 1.51] | | | | 0.18 [-0.29, 0.65] | | | 1.21 [0.74, 1.98] | | | |  |  |  |
| **Model 6** | **Final model - Patient, socioeconomic, surgical, unit characteristics with interactions** | **0.86 [0.63, 1.17]** | | **1.03 [0.70, 1.51]** | | | | **0.17 [-0.30, 0.65]** | | | **1.20 [0.74, 1.96]** | | | |  |  |  |
| Sensitivity 1 | Base, including cases with missing data | 0.88 [0.71, 1.08] | | 1.03 [0.70, 1.50] | | | | 0.15 [-0.28, 0.58] | | | 1.17 [0.76, 1.81] | | | |  |  |  |
| Sensitivity 2 | Unit and surgeon clustering (multilevel logistic regression model) | - |  | - | | |  | | - | | | | 1.07* [0.67, 1.72] | | | |  |
|  |  |  |  |  | | |  | |  | | | |  | | | |  |
| *One-year reoperation* | |  |  |  | | |  | |  | | | |  | | | |  |
| Model 1 | Base | 2.63 [2.31, 2.98] | | 1.98 [1.48, 2.64] | | | | -0.65 [-1.31, 0.01] | | | 0.75 [0.55, 1.03] | | | |  |  |  |
| Model 2 | Patient characteristics | 2.47 [2.17, 2.82] | | 1.97 [1.47, 2.63] | | | | -0.51 [-1.16, 0.15] | | | 0.80 [0.58, 1.09] | | | |  |  |  |
| Model 3 | Patient, socioeconomic characteristics | 2.49 [2.19, 2.84] | | 1.97 [1.47, 2.63] | | | | -0.52 [-1.18, 0.13] | | | 0.79 [0.58, 1.08] | | | |  |  |  |
| Model 4 | Patient, socioeconomic, surgical characteristics | 2.32 [2.00, 2.68] | | 1.95 [1.46, 2.60] | | | | -0.37 [-1.03, 0.29] | | | 0.84 [0.61, 1.16] | | | |  |  |  |
| Model 5 | Patient, socioeconomic, surgical, unit characteristics | 2.21 [1.79, 2.73] | | 1.96 [1.46, 2.62] | | | | -0.25 [-0.98, 0.47] | | | 0.89 [0.62, 1.26] | | | |  |  |  |
| **Model 6** | **Final model - Patient, socioeconomic, surgical, unit characteristics with interactions** | **2.30 [1.84, 2.87]** | | **1.79 [1.34, 2.39]** | | | | **-0.51 [-1.23, 0.21]** | | | **0.78 [0.54, 1.12]** | | | |  |  |  |
| Sensitivity 1 | Base, including cases with missing data | 2.66 [2.35, 3.02] | | 1.97 [1.48, 2.63] | | | | -0.69 [-1.35, -0.03] | | | 0.74 [0.54, 1.01] | | | |  |  |  |
| Sensitivity 2 | Unit and surgeon clustering (multilevel logistic regression model) | - |  | - | | |  | | - | | | | 0.75* [0.53, 1.06] | | | |  |

Figure S21 - Summary of flexible parametric survival model building for UKR. Estimates were generated using standardised survival functions, with covariates standardised to those of day case patients. See Table 5 for details of the individual variables comprising each covariate group. *=Odds ratio rather than RR.


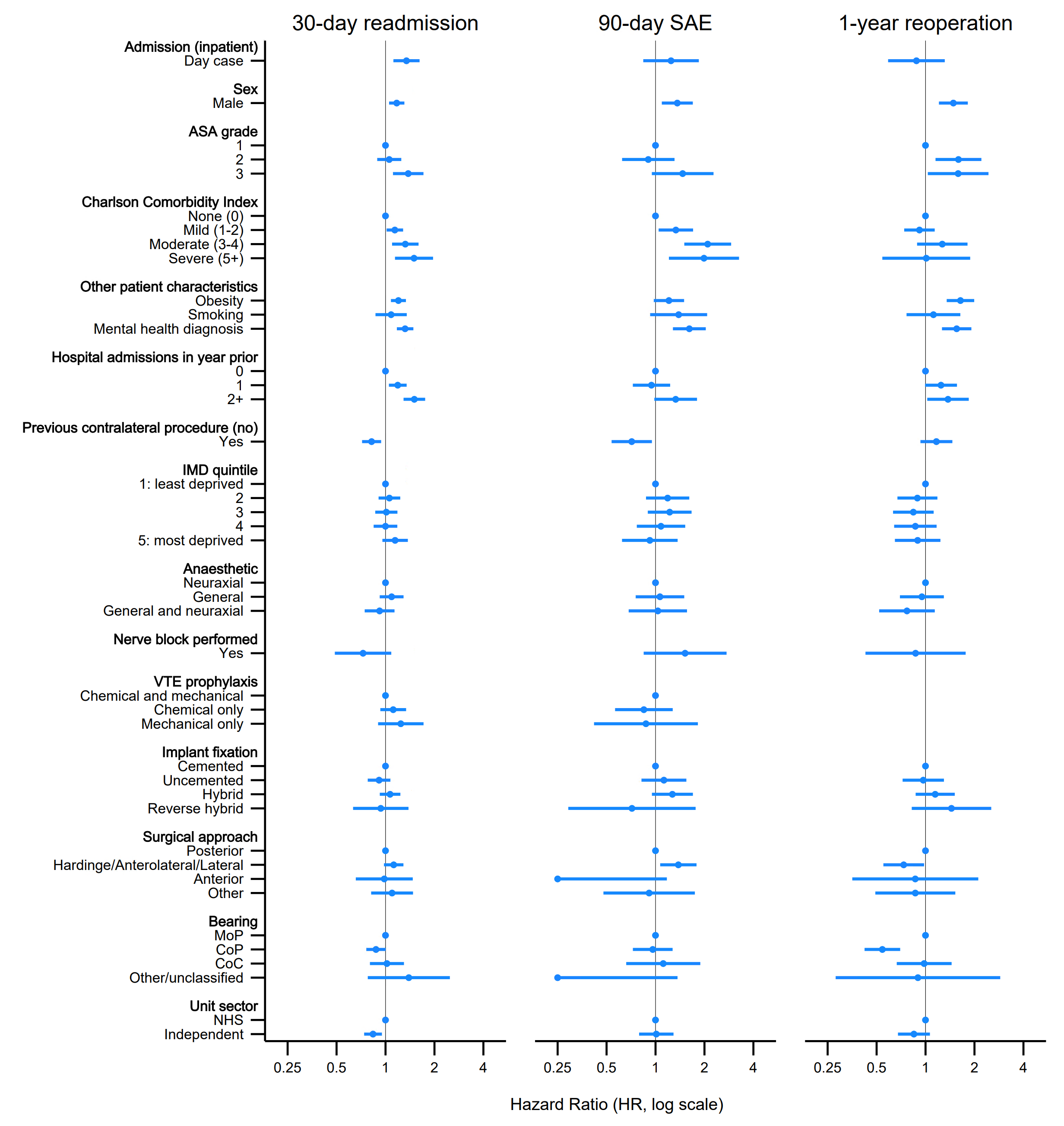


Figure S22 – Forest plot showing categorical risk factors for each outcome following THR for both day cases and inpatients (LOS 1), rather than specific risk factors for day case as shown in the main text. Generated from flexible parametric models without interaction terms (model 5 – see supplementary figure 12), and continuous variables (not shown here, see next figure) modelled using restricted cubic splines centered on median values. Bars represent 95% confidence intervals which have been truncated at <0.25 and >4 to aid readability. SAE = Serious Adverse Event; ASA = American Society of Anaesthesiologists grade; IMD = Index of Multiple Deprivation; VTEP = Venous Thromboembolism Prophylaxis; CoP = Ceramic-on-polyethylene; MoP = Metal-on-polyethylene; CoC = Ceramic-on-ceramic.


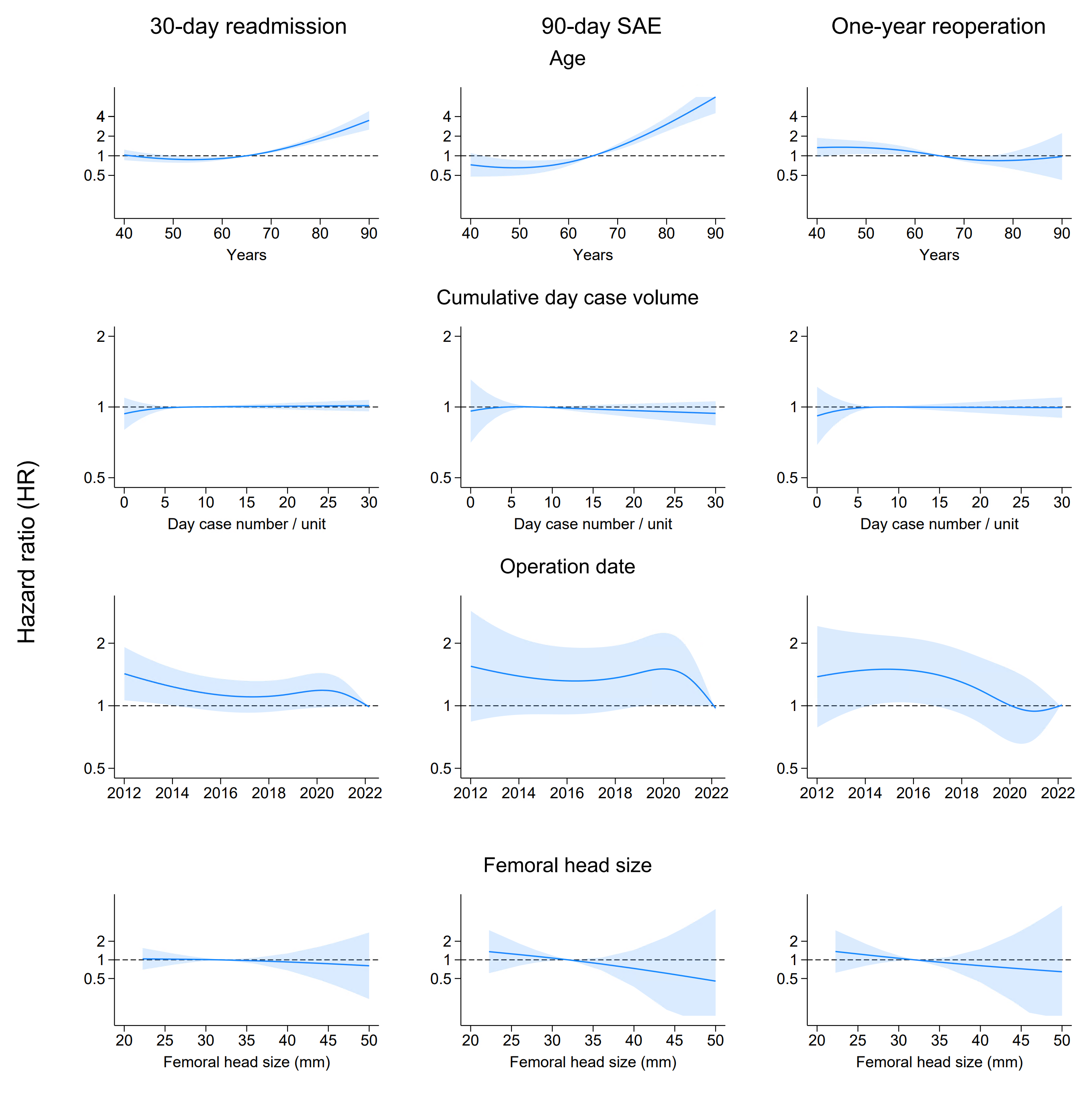


Figure S23 - Continuous risk factors for each outcome following THR for both day cases and inpatients (LOS 1), rather than specific risk factors for day case as shown in the main text. Generated from flexible parametric models without interaction terms (model 5 – see figure 12) with continuous variables modelled using restricted cubic splines centered on median values, aside from operation date which was centered on 1^st^ January 2022. Shaded areas represent 95% confidence intervals. SAE = Serious Adverse Event.


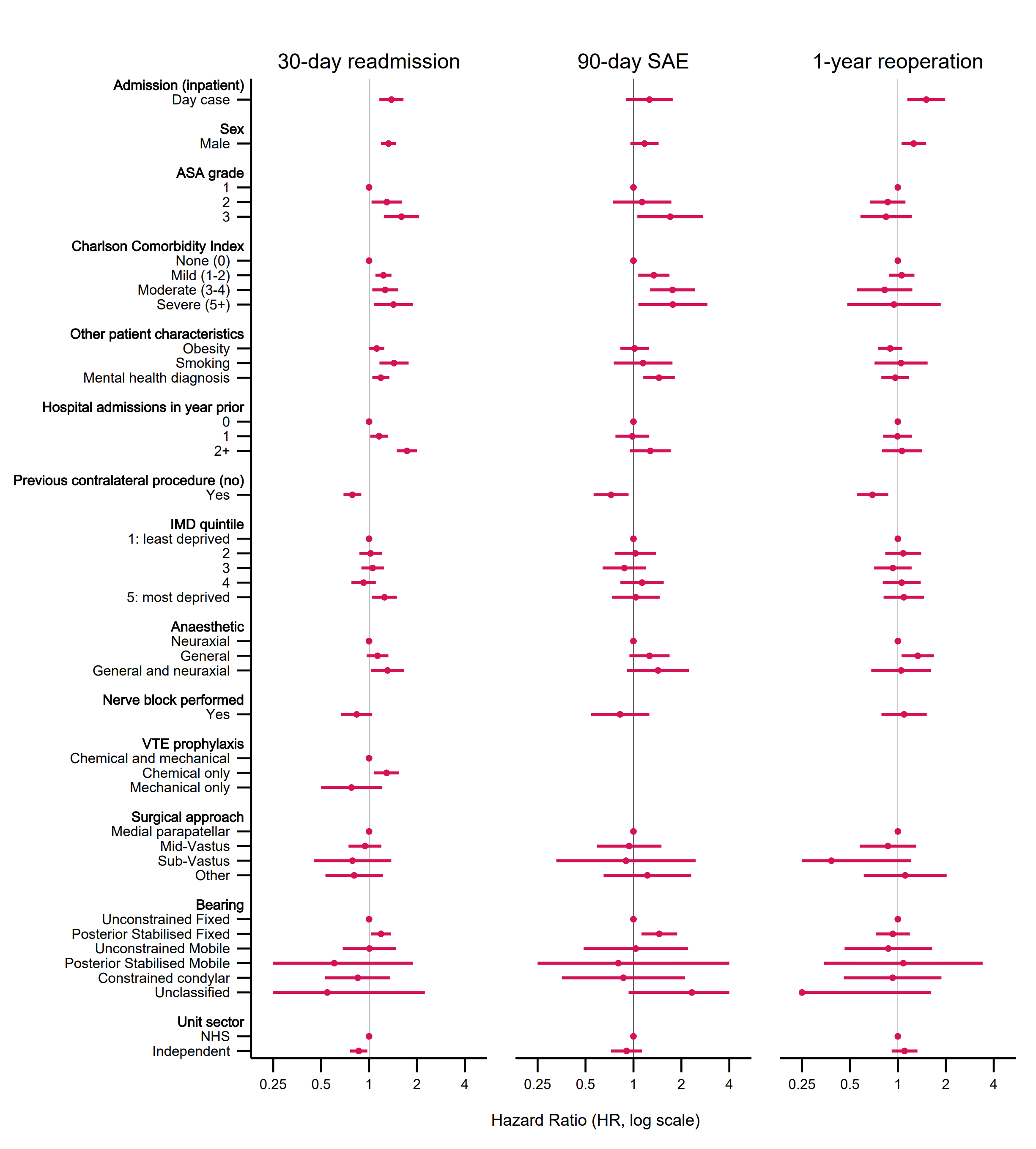


Figure S24 - Forest plot showing categorical risk factors for each outcome following TKR for both day cases and inpatients (LOS 1), rather than specific risk factors for day case as shown in the main text. Generated from flexible parametric models without interaction terms (model 5 – see supplementary figure 12), and continuous variables (not shown here, see next figure) modelled using restricted cubic splines centred on median values. Bars represent 95% confidence intervals which have been truncated at <0.25 and >4 to aid readability. SAE = Serious Adverse Event; ASA = American Society of Anaesthesiologists grade; IMD = Index of Multiple Deprivation; VTE = Venous Thromboembolism.


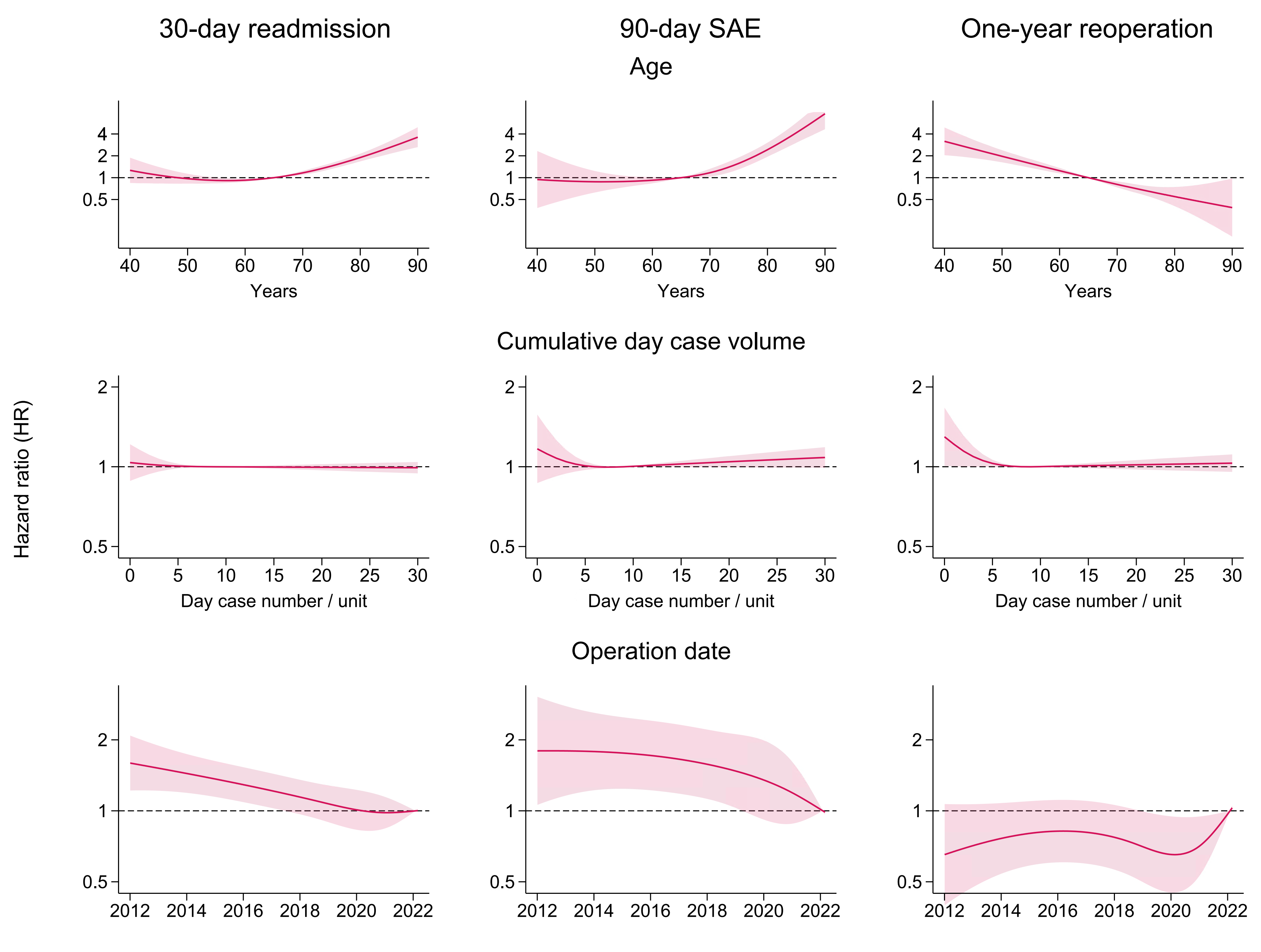


Figure S25 - Continuous risk factors for each outcome following TKR for both day cases and inpatients (LOS 1), rather than specific risk factors for day case as shown in the main text. Generated from flexible parametric models without interaction terms (model 5 – see figure 12) with continuous variables modelled using restricted cubic splines centered on median values, aside from operation date which was centered on 1^st^ January 2022. Shaded areas represent 95% confidence intervals. SAE = Serious Adverse Event.


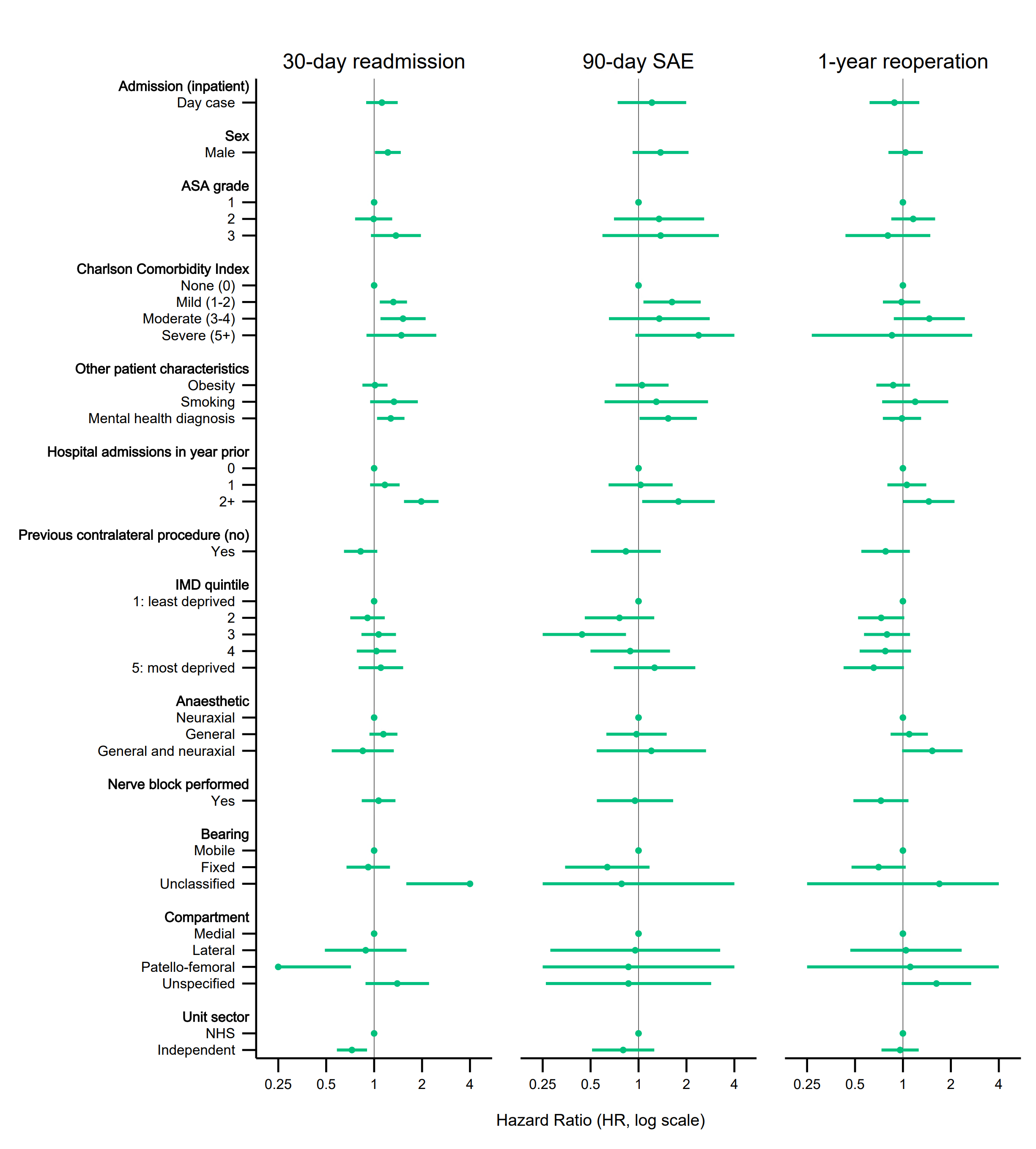


Figure S26 - Forest plot showing categorical risk factors for each outcome following UKR for both day cases and inpatients (LOS 1), rather than specific risk factors for day case as shown in the main text. Generated from flexible parametric models without interaction terms (model 5 – see supplementary figure 12), and continuous variables (not shown here, see next figure) modelled using restricted cubic splines centered on median values. Bars represent 95% confidence intervals which have been truncated at <0.25 and >4 to aid readability. SAE = Serious Adverse Event; ASA = American Society of Anaesthesiologists grade; IMD = Index of Multiple Deprivation; VTE = Venous Thromboembolism.


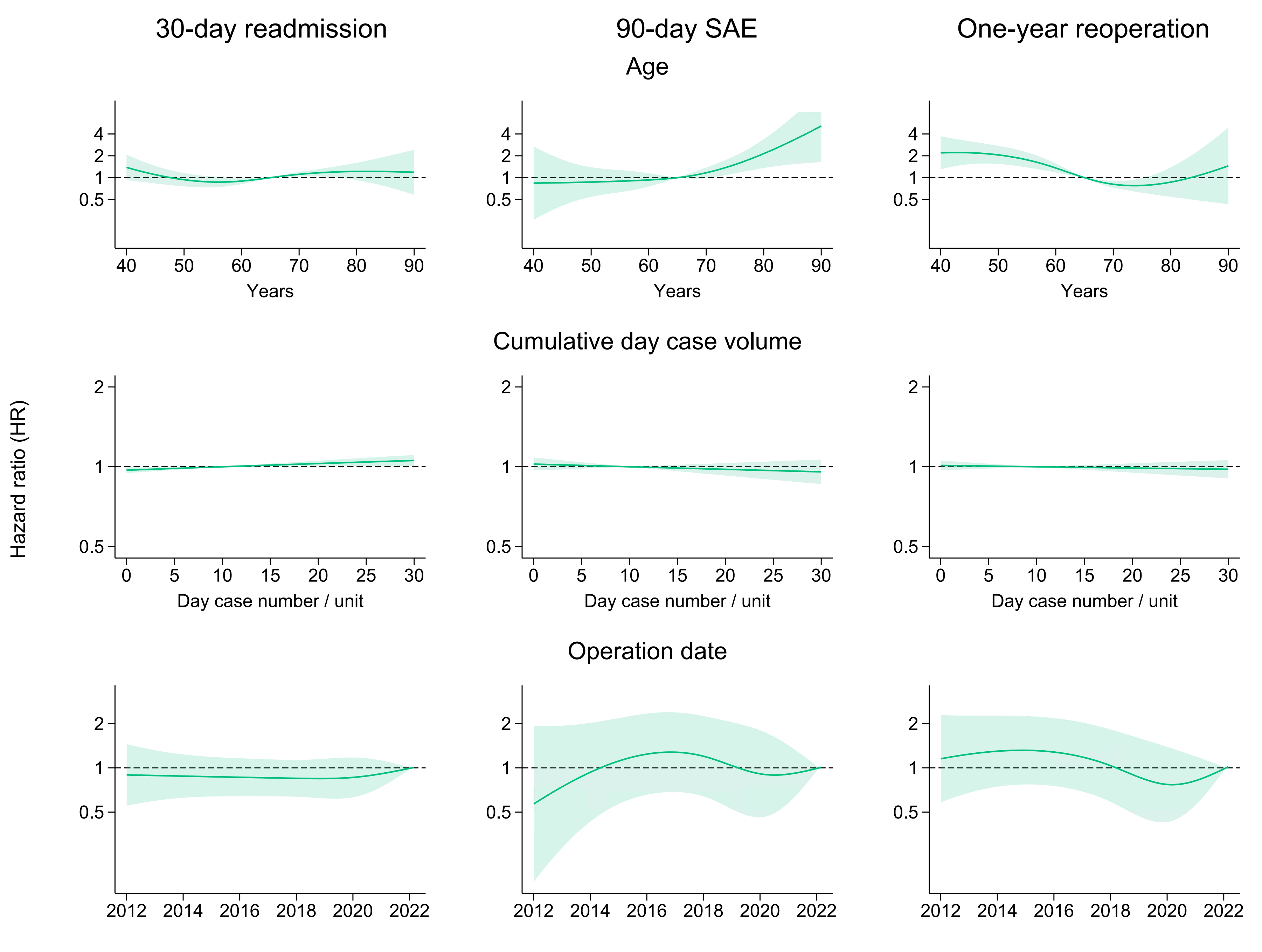


Figure S27 - Continuous risk factors for each outcome following UKR for both day cases and inpatients (LOS 1), rather than specific risk factors for day case as shown in the main text. Generated from flexible parametric models without interaction terms (model 5 – see figure 12) with continuous variables modelled using restricted cubic splines centered on median values, aside from operation date which was centered on 1^st^ January 2022. Shaded areas represent 95% confidence intervals. SAE = Serious Adverse Event.


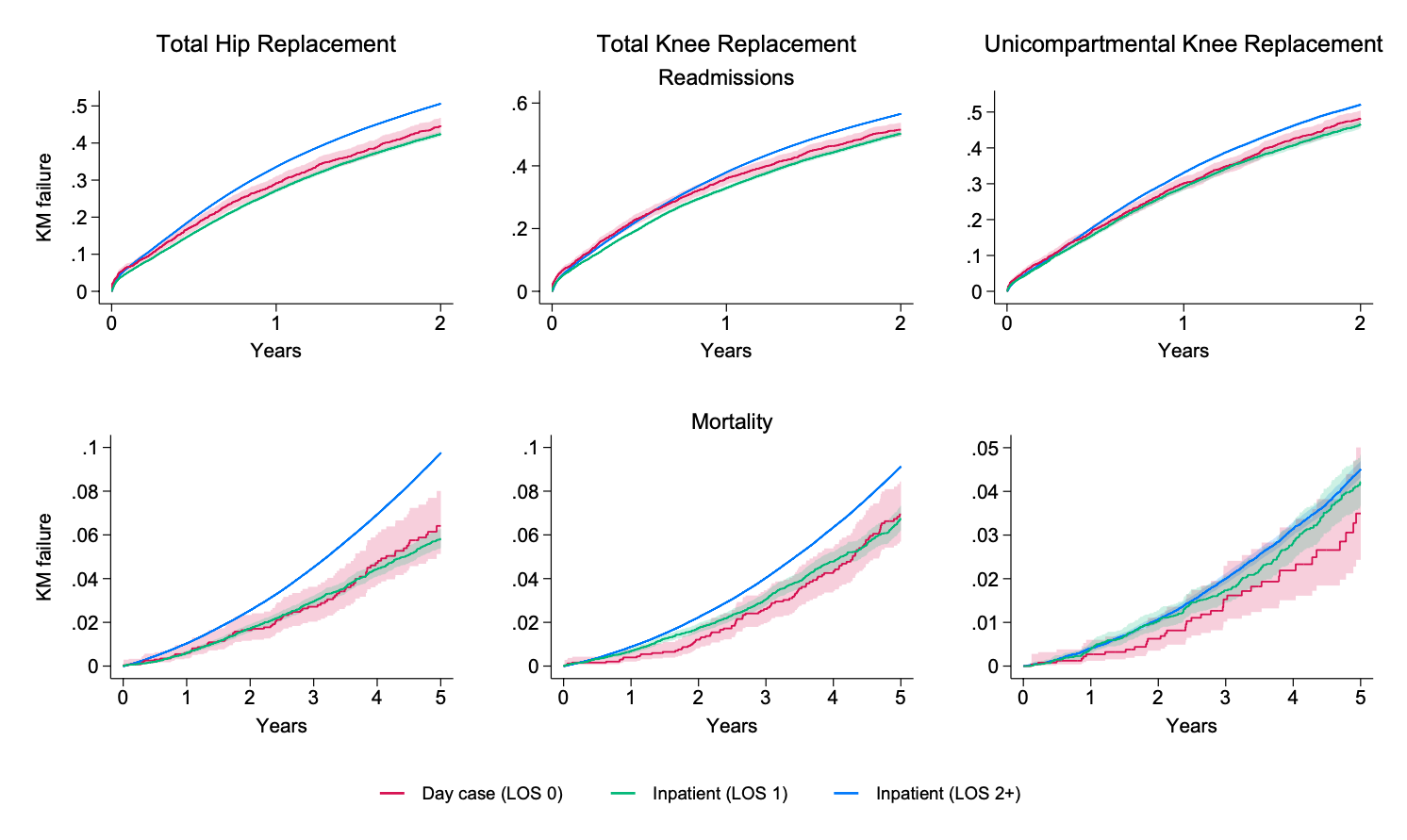


Figure S28 – Crude Kaplan-Meier failure curves showing longer-term readmission and mortality rates, stratified by length of stay (LOS). The time periods of 2 and 5 years respectively cover far beyond where any effect of a day case procedure would reasonably be expected to end. This validated the use of one-day inpatient cases, rather than all inpatients regardless of length of stay, as the comparison group; the baseline readmission and mortality hazards for LOS 0 and LOS 1 patients are well matched, but are significantly different compared to all inpatients. Shaded area = 95% confidence intervals.
